# Integrated biochemical, ionic and ultrastructural analysis of salinity tolerance in novel citrus rootstocks

**DOI:** 10.1186/s12870-026-08999-5

**Published:** 2026-05-28

**Authors:** Aditya Dnyaneshwar Ingole, Radha Mohan Sharma, Nimisha Sharma, Amit Kumar Goswami, Ajay Kumar, Sudhir Kumar, Shailendra Jha, Anil Kumar Dubey

**Affiliations:** 1 Division of Fruits and Horticultural Technology, ICAR-Indian Agricultural Research Institute, New Delhi, 110012 India; 2 Division of Plant Physiology, ICAR-Indian Agricultural Research Institute, New Delhi, 110012 India; 3 Division of Genetics, ICAR-Indian Agricultural Research Institute, New Delhi, 110012 India; 4 ICAR-Central Soil Salinity Research Institute (CSSRI), Regional Research Station, Old Jail Road, PO Dikusha, Lucknow, 226002 India

**Keywords:** Salinity, Citrus, Osmolytes, Antioxidants, Homeostasis, Ultrastructure, TEM

## Abstract

**Background:**

Salinity stress is a major constraint to citrus productivity, which is adversely affecting physiological, biochemical and cellular processes. The use of salt-tolerant rootstocks offers a sustainable strategy to mitigate these effects; however, rootstock breeding requires a comprehensive understanding of genotype-specific tolerance mechanisms operating across multiple biological levels.

**Methods:**

Six citrus rootstocks (X9, JK, S19, C9, C13 and S12) were evaluated under control and 50 mM NaCl conditions. A comprehensive assessment included gas exchange traits (*A*,* g*_*s*_, *C*_*i*_ and E), photosynthetic pigments, oxidative stress markers (H₂O₂ and MDA), antioxidant enzyme activities (SOD, APOX, CAT, GR and POX), osmolytes (LPC, TSS and TSP), TPh and mineral ion composition (Na⁺, Cl⁻, K⁺, Ca²⁺, and Mg²⁺) in leaves and roots. Chloroplast ultrastructure was analyzed using transmission electron microscopy. Multivariate analyses, including principal component analysis, hierarchical clustering and membership function value analysis, were used to establish a salinity tolerance ranking.

**Results:**

Salinity significantly affected all traits, with pronounced genotype-dependent variation. Under the present experimental conditions, the tolerance ranking was observed as: X9 > C13> S12 > S19 > C9 > JK. This hierarchy was supported by photosynthetic rate as tolerant genotypes exhibited comparatively lower reductions in rate (44–51%) than sensitive genotypes (62–86%), with X9 showing the least decline (44.3%). Chlorophyll loss ranged from 4.3% to 19.7%, in parallel with photosynthetic performance. Oxidative damage was substantially lower in tolerant genotypes, as indicated by reduced increases in H₂O₂ (8.1–83.3%) and MDA (31.9–86.6%). Enhanced antioxidant responses (SOD, APOX and GR) and greater accumulation of osmolytes and phenolics contributed to stress mitigation. X9 maintained the highest K⁺/Na⁺ ratio (4.5) through effective exclusion of Na⁺ and Cl⁻, while sensitive genotypes accumulated toxic levels. Ultrastructural analysis confirmed preservation of chloroplast integrity in tolerant genotypes, in contrast to severe structural damage in sensitive ones. Principal component analysis clearly distinguished tolerant and sensitive groups.

**Conclusions:**

Salinity tolerance in citrus rootstocks is controlled by the coordinated integration of ion regulation, antioxidant defense, osmotic adjustment, photosynthetic maintenance and chloroplast integrity. X9 emerged as the most tolerant genotype, followed by C13 and S12. The multi-trait framework and quantitative analysis identified here provide practical criteria for breeding and selection of citrus rootstocks for salt-affected environments.

## Introduction

Citrus is a major commercial fruit crop cultivated globally, with an annual production exceeding 160 million tons [1]. India contributes approximately 8% of the world’s citrus production [1], thus playing a crucial role. Rich in essential nutrients like vitamin C, dietary fiber and potassium, citrus fruits are also valued for their therapeutic properties. They contain diverse phytochemicals, such as flavonoids and phenolic compounds, which possess strong antioxidant qualities [2–6]. Under a changing climate, the sustainability of citrus production is threatened by soil salinization, a major abiotic stress that limits crop productivity worldwide [7, 8]. This problem is particularly severe in arid and semi-arid regions, where the use of saline irrigation water and inadequate drainage accelerate salt accumulation in the upper layers of the soil [9, 10]. Salinity induces combined osmotic and ionic stress, which primarily limits water uptake [11]. Consequently, it impairs photosynthesis and disrupts metabolic processes and ionic homeostasis [8, 12–15]. Excessive accumulation of sodium (Na⁺) and chloride (Cl⁻) ions within plant tissues causes direct ionic toxicity. Furthermore, these ions competitively inhibit the uptake of essential cations like potassium (K⁺), calcium (Ca²⁺) and magnesium (Mg²⁺), thereby reducing membrane stability and enzymatic activity [7, 12, 16]. The maintenance of a high K⁺/Na⁺ ratio is considered a key indicator of salt tolerance, reflecting a capacity of genotype to regulate ion uptake and preserve ionic homeostasis under salt stress [15, 17].

Salinity induces severe oxidative stress in plants through the excessive production of reactive oxygen species (ROS), such as superoxide radicals (O₂⁻) and hydrogen peroxide (H₂O₂), which leads to lipid peroxidation, protein degradation and nucleic acid damage [18, 19]. Malondialdehyde (MDA), a major end product of lipid peroxidation, is widely used as a reliable biomarker of membrane injury under salt stress [20]. To counteract oxidative damage, plants activate a coordinated antioxidant defense system comprising enzymes such as superoxide dismutase (SOD), catalase (CAT), ascorbate peroxidase (APOX), glutathione reductase (GR) and peroxidase (POX). Together, these enzymes play a crucial role in maintaining cellular redox homeostasis [21–23]. In parallel, osmotic adjustment is achieved through the accumulation of compatible solutes, including proline, total soluble sugars and total soluble proteins, which stabilize cellular membranes, protect enzymes and maintain turgor under saline conditions [24–26]. Additionally, secondary metabolites, particularly phenolic compounds, play a key role in stress mitigation by acting as antioxidants and scavenging reactive oxygen species [27]. The magnitude and coordination of these antioxidant and osmotic responses ultimately determine the level of genotypic tolerance to salinity stress [28].

Rootstocks play a crucial role in conferring salinity tolerance by regulating ion uptake, transport and distribution, thereby protecting the scion from ionic toxicity [29]. Salt-tolerant rootstocks effectively restrict the accumulation of Na⁺ and Cl⁻ while maintaining adequate levels of essential nutrients, particularly K⁺ and Ca²⁺ [30, 31]. This rootstock-mediated tolerance is closely associated with enhanced antioxidant enzyme activity and improved osmotic regulation, which together contribute to sustained cellular homeostasis under saline conditions [32, 33].

Although multi-level assessments exist, studies integrating ionic homeostasis (Na⁺/K⁺ balance) with direct visualization of chloroplast ultrastructure via transmission electron microscopy (TEM) within a single experimental framework remain scarce, limiting our understanding of the structural consequences of NaCl-induced ion toxicity in citrus rootstocks. Since 2008, the Division of Fruits and Horticultural Technology at ICAR–IARI, New Delhi, has developed promising polyembryonic citrus rootstock hybrids. The primary objectives of this program were to combine polyembryony with enhanced salinity tolerance in citrus rootstocks. Polyembryony is a critical trait for rootstock propagation in citrus, as it enables the production of uniform, true-to-type seedlings via seed. Still, the mechanisms of their salinity tolerance are relatively unknown. To address this gap, this study evaluates integrated biochemical, enzymatic and ionic responses of these novel interspecific and intergeneric hybrids to NaCl stress, focusing on osmolyte accumulation, antioxidant enzyme activity and ionic regulation to identify salt-resilient rootstocks for saline environments.

## Materials and methods

### Plant material

The study was conducted at the experimental orchard of the Division of Fruits and Horticultural Technology, ICAR–Indian Agricultural Research Institute (IARI), New Delhi (28°40′ N latitude, 77°12′ E longitude, elevation 228.6 m above mean sea level). Six citrus rootstocks, including four newly developed polyembryonic hybrids (Table 1), were raised in a shade house starting in November 2022. After six months, healthy and uniform seedlings were transplanted into 30 cm diameter pots containing a 3:1:1 mixture of topsoil, farmyard manure and vermicompost. Plants were allowed to establish for six months in pots under optimal conditions. During the experiment, plants were grown under natural photoperiod conditions in a greenhouse. Photosynthetic photon flux density (PPFD) ranged from 800 to 1,200 µmol m⁻² s⁻¹ at midday, measured using a quantum sensor (LI-190R, LI-COR, Lincoln, NE, USA). Day/night temperatures averaged 32 ± 4 °C and 22 ± 3 °C, respectively, during the experimental period. Relative humidity ranged from 45% to 70%. During the establishment period, plants were irrigated regularly with tap water (pH 7.1) to field capacity and received a weekly foliar spray of NPK (19:19:19) to support uniform growth. To avoid confounding effects on salinity responses, the foliar NPK application was discontinued one month before the initiation of salt treatment and was not applied during the experimental period. This ensured that all measured responses during the salinity stress phase reflected genotype-specific tolerance mechanisms rather than differential fertilizer effects. The experiment was arranged in a factorial completely randomized design (FCRD) with two factors: six citrus and two salinity levels (control and 50 mM NaCl). Each treatment combination was replicated three times, with three plants per replication.

**Table 1:** List of the genotypes utilized in the study

Sr.No.	Citrus genotypes	Scientific name/Parentage
1	SCSH17-12 (S12)	*C.maxima* Merr.×*C.sinensis* (L.) Osbeck
2	SCSH9-19 (S19)	*C.maxima* Merr.×*C.sinensis* (L.) Osbeck
3	CRH21-13(C13)	*C.maxima*Merr.×Troyer[(*C.sinensis* (L.) Osbeck×*P. trifoliate* (L.) Raf.)]
4	CRH21-9 (C9)	*C.maxima* Merr.×Troyer[(*C.sinensis* (L.) Osbeck× *P.trifoliate* (L.))]
5	X-639 (X9)	*C.reshni* hort.ex Tanaka× *P.trifoliate* (L.)
6	Jatti Khatti (JK)	*C. jambhiri* Lush.

### Details of salinity treatment

Salinity stress was induced by irrigating plants with a 50 mM sodium chloride (NaCl) solution, prepared by dissolving 2.92 g of NaCl per liter of tap water, while control plants received tap water only (EC = 0.28 dS m⁻¹). The treatment was applied over 42 days, with each pot receiving 3.3 L of the respective irrigation solution per watering event to maintain soil moisture at field capacity. A total of six irrigations were administered, which significantly increased soil salinity, the electrical conductivity (EC 1:2) rose from an initial 0.31 dS m⁻¹ to a final 4.20 dS m⁻¹. Salt stress was maintained until the sensitive reference genotype, JK, exhibited visible symptoms of salt injury (leaf chlorosis and necrosis). At this point, final irrigation was applied. After a week of last irrigation, mature leaf samples were harvested, rapidly frozen in liquid nitrogen and stored at -80 °C to preserve biochemical, enzymatic and ionic integrity for subsequent analysis.

### Physiological observations

Chlorophyll fractions [chlorophyll a (Chl a), chlorophyll b (Chl b) and total chlorophyll (Tchl)] were quantified using the dimethyl sulfoxide (DMSO) method, with absorbance measured at 665 nm and 648 nm using a UV–VIS spectrophotometer (UV-1900i; Shimadzu). Leaf gas exchange parameters—net photosynthesis (*A*, µmol m⁻² s⁻¹), stomatal conductance (*gₛ*, mmol m⁻² s⁻¹), intercellular CO₂ concentration (*Ci*, µmol mol⁻¹) and transpiration rate (*E*, mmol H₂O m⁻² s⁻¹)—were recorded with an LCpro-SD portable infrared gas analyzer.

### Biochemical and enzymatic assays

#### ROS activity (mmol g-1 FW)

##### Hydrogen peroxide (H₂O₂)

The H₂O₂ content was quantified following the method described by Patterson [34]. Fresh leaf tissue (1 g) was homogenized in 2 mL of acetone, and the homogenate was centrifuged to obtain the supernatant. Titanium reagent was added to the supernatant, followed by the addition of 17 M ammonia solution to precipitate the peroxide–titanium complex. The resulting precipitate was separated, washed thoroughly with acetone and subsequently dissolved in 3 mL of 2 N sulfuric acid (H₂SO₄). Absorbance was measured at 410 nm using a PerkinElmer Lambda 365 UV–Vis spectrophotometer. A reagent blank, prepared identically without plant tissue, was used as the reference.

### Osmolyte and protein quantification

#### Total Soluble Sugars (TSS)

The TSS were extracted from 0.1 g of fresh leaf tissue using 5 mL of 80% ethanol. The extract was centrifuged at 10,000 × g for 10 min, and the supernatant was reacted with an equal volume of anthrone reagent following Yemm and Willis [35]. The reaction mixture was incubated in a boiling water bath for 10 min and then cooled to room temperature. Absorbance was recorded at 620 nm against a reagent blank. Sugar concentration was determined using a glucose standard curve and expressed as mg g⁻¹ fresh weight (FW).

#### Leaf Proline Content (LPC)

The LPC was estimated according to the method of Bates [36]. Briefly, 0.5 g of fresh leaf tissue was homogenized in 10 mL of 3% (w/v) aqueous sulfosalicylic acid and filtered. An aliquot of 2 mL of the filtrate was reacted with 2 mL of acid ninhydrin and 2 mL of glacial acetic acid. The mixture was incubated at 100 °C for 1 h, and the reaction was terminated by placing the tubes in an ice bath. The chromophore was extracted with 4 mL of toluene, and the absorbance was measured at 520 nm. The LPC concentration was calculated from a standard curve and expressed as µg g⁻¹ FW.

#### Total Soluble Protein (TSP)

The TSP content was determined using the Bradford [37] method. An aliquot of the leaf extract, prepared in an appropriate buffer, was mixed with Bradford reagent, and absorbance was measured at 595 nm after a 5-min incubation. Protein concentration was calculated using bovine serum albumin (BSA) as the standard and expressed as mg g⁻¹ FW.

### Lipid peroxidation and phenolic compounds

#### Total phenolic (TPh)

The TPh content was determined using the Folin–Ciocalteu reagent following Singleton and Rossi [38]. An aliquot of 0.1 mL of the leaf extract was mixed with 0.5 mL of Folin–Ciocalteu reagent (diluted 1:10, v/v) and 1.5 mL of sodium carbonate solution (20%, w/v). The reaction mixture was vortexed and incubated in the dark at room temperature for 30 min. Absorbance was measured at 765 nm. The TPh content was expressed as mg gallic acid equivalents (GAE) g⁻¹ fresh weight (FW), calculated using a gallic acid standard curve.

#### Malondialdehyde (MDA) estimation

MDA content was quantified by estimating thiobarbituric acid reactive substances (TBARS) according to Heath and Packer [39]. Fresh leaf tissue (0.5 g) was homogenized in 5 mL of 0.1% (w/v) trichloroacetic acid (TCA) and centrifuged at 10,000 × g for 10 min. An aliquot of 1 mL of the supernatant was mixed with 4 mL of 0.5% (w/v) thiobarbituric acid (TBA) prepared in 20% (w/v) TCA. The mixture was heated at 95 °C for 30 min, rapidly cooled in an ice bath and centrifuged again. Absorbance of the supernatant was recorded at 532 nm and non-specific turbidity was corrected by subtracting absorbance at 600 nm. MDA concentration was calculated using an extinction coefficient of 155 mM⁻¹ cm⁻¹ and expressed as µM MDA g⁻¹ FW.

### Antioxidant enzyme activities

#### Enzyme extraction

All enzymes were extracted from 0.5 g of fresh leaf tissue homogenized in 5 mL of ice-cold 50 mM potassium phosphate buffer (pH 7.8) containing 1 mM EDTA, 1% (w/v) polyvinylpyrrolidone (PVP) and 1 mM phenylmethylsulfonyl fluoride (PMSF). The homogenate was centrifuged at 15,000 × g for 20 min at 4 °C and the resulting supernatant was used as the crude enzyme extract.

#### SOD

The SOD (EC 1.15.1.1) activity was assayed based on its ability to inhibit the photochemical reduction of nitroblue tetrazolium (NBT) as described by Fridovich [40]. The 3 mL reaction mixture comprised 50 mM phosphate buffer (pH 7.8), 13 mM methionine, 75 µM NBT, 2 µM riboflavin, 0.1 mM EDTA and an appropriate aliquot of the enzyme extract. Following a 15-minute illumination under a fluorescent lamp, absorbance was recorded at 560 nm. One unit of SOD activity was defined as the amount of enzyme required to inhibit 50% of the NBT reduction.

#### APOX

The APOX (EC 1.11.1.11) activity was determined by monitoring the oxidation of ascorbate at 290 nm (ε = 2.8 mM⁻¹ cm⁻¹) according to Nakano and Asada [41]. The 1 mL reaction mixture contained 50 mM potassium phosphate buffer (pH 7.0), 0.5 mM ascorbate, 0.1 mM H₂O₂ and enzyme extract. The reaction was initiated by the addition of H₂O₂.

#### CAT

The CAT (EC 1.11.1.6) activity was measured by the method of Aebi [42] through the decrease in absorbance at 240 nm (ε = 39.4 M⁻¹ cm⁻¹) due to the decomposition of H₂O₂. The assay mixture (50 mM phosphate buffer, pH 7.0, containing 15 mM H₂O₂ and enzyme extract) was monitored for 1 min. One unit of activity was defined as the amount of enzyme decomposing 1 µmol of H₂O₂ per minute.

#### GR

The GR (EC 1.8.1.7) activity was assayed by following the oxidation of NADPH at 340 nm (ε = 6.22 mM⁻¹ cm⁻¹) as described by Smith [43]. The reaction mixture consisted of 0.1 M phosphate buffer (pH 7.8), 1 mM EDTA, 1 mM oxidized glutathione (GSSG), 0.1 mM NADPH and enzyme extract.

#### POX

The POX (EC 1.11.1.7) activity was evaluated using guaiacol as the substrate [44]. Activity was determined by monitoring the increase in absorbance at 470 nm due to tetraguaiacol formation (ε = 26.6 mM⁻¹ cm⁻¹). The 3 mL reaction mixture contained 50 mM phosphate buffer (pH 6.5), 10 mM guaiacol, 5 mM H₂O₂ and enzyme extract.

### Leaf and root nutrient analysis

Mature leaf samples were harvested and sequentially washed with tap water, 0.2% Teepol solution, 0.1 N HCl and double-distilled water to remove surface contaminants. The leaves were then oven-dried at 70 °C for 48 h and ground to a fine powder using a Wiley mill. The powder was passed through a 1 mm mesh sieve and stored in airtight containers until analysis.

After harvesting, root systems were gently washed with deionized water to remove adhering soil particles. To eliminate surface-bound contaminants and apoplastic ions, the roots were sequentially washed in (i) 0.2% (v/v) Teepol solution, (ii) 0.1 M HCl for 30 s and (iii) three successive rinses with ultrapure water (Milli-Q). The cleaned root tissues were then blotted dry using paper towels. The samples were placed in paper envelopes and dried in a forced-air oven at 70 °C until a constant weight was obtained for 72 h. The dried samples were then ground into a fine powder using a stainless steel centrifugal mill fitted with a 1.0 mm sieve. The powdered samples were stored in sterile polypropylene vials under dry and dark conditions until further analysis.

### Sample digestion for mineral analysis

For the quantification of potassium (K⁺), sodium (Na⁺), calcium (Ca²⁺) and magnesium (Mg²⁺), a 0.5 g aliquot of the powdered leaf and root material was subjected to nitric acid digestion. The sample was pre-digested overnight in 10 mL of concentrated nitric acid (HNO₃). Subsequently, it was digested on a hot plate, first at 100 °C for 1 h, then at 200 °C for 2–3 h, until the solution became colorless and dense white fumes ceased. The resulting digest was cooled, diluted with double-distilled water, filtered through Whatman No. 1 filter paper and made up to a final volume of 50 mL in a volumetric flask.

### Elemental analysis

#### Potassium and sodium

Concentrations of K⁺ and Na⁺ in the digest were determined using a microprocessor-based flame photometer (Systronics Flame Photometer-128, Ahmedabad, India). Appropriately diluted samples were aspirated and concentrations were read directly. Results were calculated by applying the dilution factor and are expressed as a percentage of dry weight (% DW) [45].

#### Calcium and magnesium

Concentrations of Ca²⁺ and Mg²⁺ were quantified using an atomic absorption spectrophotometer (GBC 904AA, GBC Scientific Equipment, USA). For calcium, the diluted digest was aspirated into a nitrous oxide–acetylene flame, and absorbance was recorded at 422 nm [45]. For magnesium, absorbance was measured at 285.2 nm using an air–acetylene flame. To suppress chemical interference, 0.1% (w/v) lanthanum oxide (La₂O₃) was added to all standards and samples. The concentrations of both elements were determined using standard calibration curves and expressed as % DW.

#### Chloride

Chloride (Cl⁻) concentration was estimated spectrophotometrically using the mercury(II) thiocyanate method (46). Chloride was extracted from 0.1 g of dried leaf powder with 0.1 M sodium nitrate at a 1:100 (w/v) ratio. The reaction mixture consisted of 10 mL of the sample extract, 2 mL of ferric(III) nitrate nanohydrate solution and 2 mL of saturated mercury(II) thiocyanate solution [46]. After a 10-minute incubation, the brilliant brick-red color formed was measured at 460 nm using a UV–Vis spectrophotometer (UVD 3200, Labomed, USA). The chloride concentration was determined from a standard calibration curve and expressed as % DW.

#### Transmission Electron Microscopy (TEM)

Leaf samples were collected from all six citrus rootstocks under both control and salinity treatments at the end of the experimental period. For each genotype and treatment, three biological replicates were analyzed. Fresh citrus leaf samples from control and stress-treated plants were immediately cut into 1–2 mm² segments. Primary fixation was performed in 2.5% glutaraldehyde (in 0.1 M phosphate buffer, pH 7.2) at 4 °C for 12–24 h. After rinsing in buffer, samples were post-fixed in 1% osmium tetroxide for 2 h at 4 °C. Tissues were dehydrated in a graded ethanol series, infiltrated with epoxy resin and polymerized at 60 °C [47]. Ultrathin Sects.  (70–90 nm) were cut using an ultramicrotome, mounted on copper grids and double-stained with uranyl acetate and lead citrate. Leaf ultrastructure was analyzed using a transmission electron microscope at the Sophisticated Analytical Instrument Facility (SAIF), AIIMS, New Delhi. Samples were examined at accelerating voltages of 40–120 kV. Images were acquired with a digital CCD camera and processed using iTEM software (Olympus Soft Imaging System, Germany).

### Quantitative analysis of ultrastructural parameters

#### Chloroplast area (µm²)

Chloroplast cross-sectional area was measured from digital micrographs using image analysis software (ImageJ, NIH, USA). For each chloroplast, the outline was traced manually using the polygon selection tool and the area was calculated by the software. Only chloroplasts with clearly defined boundaries and visible internal structures were included in the analysis. Results were expressed in square micrometers (µm²).

#### Starch grain number

The number of starch grains within each chloroplast was counted manually from digital micrographs. Starch grains were identified as electron-translucent (white) oval or spherical structures within the chloroplast stroma. Only starch grains with clearly defined boundaries were counted. Results were expressed as mean number of starch grains per chloroplast section.

### Statistical analysis

All quantitative data were subjected to analysis of variance (ANOVA) using a factorial completely randomized design (FCRD) with three replications to assess the effects of genotype, salinity treatment and their interaction. Data were analyzed using two-way ANOVA with genotype and salinity as fixed factors. All results are presented as mean ± standard error (SE) of three independent replications. Statistical analyses were performed using SAS software (version 9.4, SAS Institute, Cary, NC, USA). Treatment means were compared using Tukey’s Honest Significant Difference (HSD) test at a significance level of *p* ≤ 0.05. Data are expressed as mean ± standard error (SE) of three independent replications. Comparative responses of citrus rootstocks under control and salinity treatments were illustrated using bar graphs to evaluate treatment-induced changes in oxidative stress indicators, osmolytes, antioxidant enzyme activities and ionic composition. Pearson correlation analysis, was performed using Python packages (sklearn, bioinfokit, seaborn, scipy, numpy, pandas and matplotlib) to identify key morpho-physiological and photosynthetic traits contributing to salinity tolerance among the tested citrus rootstocks. Additionally, PCA was performed on standardized trait values from combined control and salinity datasets. The first two principal components were extracted and genotype scores were plotted to visualize treatment separation and genotypic variation. Trait loadings were overlaid as vectors to identify key contributing traits.Salinity response patterns were visualized using hierarchical clustering of standardized trait values with Euclidean distance and Ward’s linkage method. To identify salinity-specific trait relationships, Pearson correlation matrices were calculated separately for control and salinity datasets. The interaction correlation matrix (Salinity - Control) was computed and visualized as a clustered heatmap, highlighting changes in trait associations induced by salinity stress.

Salinity tolerance of citrus rootstocks was evaluated using the Membership Function Value (MFV) method and principal component analysis (PCA). For MFV, all measured traits were first categorized based on their contribution to tolerance: beneficial traits (gas exchange parameters, osmolytes, antioxidant enzymes and essential ions) and stress-related traits (H₂O₂, MDA and toxic ions). Trait values were normalized using standard membership functions, where positively associated indicators were scaled as (x − min)/(max − min) and negatively associated indicators as 1 − (x − min)/(max − min). A comprehensive tolerance index (D value) was calculated as the mean of all membership values for each genotype and genotypes were ranked accordingly. For PCA, all variables were standardized prior to analysis. Principal components were extracted and a composite score was calculated using a weighted sum of the first two principal components based on their explained variance. Genotypes were ranked according to these PCA scores to validate the MFV-based classification.

## Results

Under control conditions, all genotypes exhibited healthy growth without visible stress symptoms (Fig. 8), whereas salinity stress induced clear genotype-dependent differences in growth and leaf injury (Fig. 9), with X9, C13 and S12 showing greater tolerance than C9, S19 and JK. The application of NaCl-induced salinity stress significantly altered the Gas Exchange traits, Photosynthetic Pigments, Reactive Oxygen Species and Lipid Peroxidation, Osmolyte and Metabolite Accumulation, Antioxidant Enzyme Activities, Leaf and root Mineral Ion Composition among six genotypes.

### Photosynthetic pigments

#### Chl a (mg g⁻¹ leaf fresh weight)

Salinity stress induced a marked decline in Chl a content across all citrus rootstocks, though the magnitude of reduction varied considerably among genotypes (Table 2; Fig. 5). Under non-saline conditions, Chl a values ranged from 0.775 ± 0.014 mg g⁻¹ FW in JK to 0.942 ± 0.017 mg g⁻¹ FW in S19, with C13 also maintaining relatively high content (0.905 ± 0.017 mg g⁻¹ FW). Exposure to salinity reduced these values to a range of 0.490 ± 0.009–0.820 ± 0.015 mg g⁻¹ FW. Notably, C13 retained the highest Chl a content under stress (0.820 ± 0.015 mg g⁻¹ FW), followed by X9 (0.760 ± 0.014 mg g⁻¹ FW) and S12 (0.700 ± 0.013 mg g⁻¹ FW). The percent reduction in Chl a ranged from a modest 8.1% in X9 to a dramatic 42.7% in S19, with substantial declines also evident in C9 (37.1%) and JK (36.8%), suggesting that these latter genotypes are particularly susceptible to salinity-induced pigment degradation.

**Table 2:** Effect of salinity stress on gas exchange parameters: net photosynthetic rate (A, µmol m⁻² s⁻¹), stomatal conductance (gₛ, mol m⁻² s⁻¹), intercellular CO₂ concentration (C_i_, µmol mol⁻¹) and transpiration rate (E, mmol H₂O m⁻² s⁻¹); photosynthetic pigments: chlorophyll *a* *(Chl a)*, chlorophyll *b(Chl b)* and total chlorophyll*(TChl)* (mg g⁻¹ FW); oxidative stress markers: H₂O₂ (µmol g⁻¹ FW) and MDA (nmol g⁻¹ FW); in six citrus rootstocks

	Chl a	Chl b	TChl	A	g_s_	Ci	E	H_2_O_2_	MDA
Control									
S12	0.826 ± 0.0152 ^bc^	0.239 ± 0.0044 ^e^	1.176 ± 0.0144 ^a^	6.81 ± 0.04 ^b^	0.0880 ± 0.0007 ^a^	152.42 ± 2.30 ^d^	1.542 ± 0.020 ^b^	44.20 ± 0.52 ^cd^	29.54 ± 0.13 ^g^
S19	0.942 ± 0.0173 ^a^	0.262 ± 0.0048 ^d^	1.175 ± 0.0144 ^a^	7.69 ± 0.05 ^a^	0.0880 ± 0.0007 ^a^	135.97 ± 3.05 ^e^	1.970 ± 0.025 ^a^	46.30 ± 0.10 ^b^	24.22 ± 0.19 ^h^
C13	0.905 ± 0.0166 ^a^	0.327 ± 0.0060 ^c^	1.064 ± 0.0130 ^c^	7.58 ± 0.05 ^a^	0.0890 ± 0.0008 ^a^	158.58 ± 2.79 ^c^	1.447 ± 0.018 ^c^	44.90 ± 0.12 ^c^	57.46 ± 0.79 ^a^
C9	0.795 ± 0.0146 ^c^	0.318 ± 0.0058 ^c^	1.139 ± 0.0139 ^ab^	6.30 ± 0.03 ^d^	0.0820 ± 0.0006 ^b^	136.66 ± 2.92 ^e^	1.525 ± 0.019 ^b^	47.60 ± 0.08 ^a^	53.22 ± 0.42 ^b^
X9	0.827 ± 0.0152 ^bc^	0.379 ± 0.0070 ^a^	1.041 ± 0.0127 ^c^	6.27 ± 0.04 ^d^	0.0900 ± 0.0011 ^a^	154.42 ± 3.30 ^cd^	1.527 ± 0.019 ^b^	44.10 ± 0.45 ^d^	37.20 ± 0.68 ^e^
JK	0.775 ± 0.0142 ^c^	0.303 ± 0.0056 ^c^	1.106 ± 0.0135 ^b^	4.81 ± 0.03 ^e^	0.0900 ± 0.0010 ^a^	134.79 ± 3.07 ^e^	1.605 ± 0.021 ^b^	47.30 ± 1.11 ^a^	45.57 ± 0.19 ^c^
Mean	0.845 ^A^	0.305 ^A^	1.117 ^A^	6.58 ^A^	0.0878 ^A^	145.47 ^B^	1.603 ^A^	45.73 ^B^	41.20 ^B^
Salinity									
S12	0.700 ± 0.0129 ^d^	0.218 ± 0.0040 ^f^	0.994 ± 0.0122 ^d^	3.32 ± 0.04 ^g^	0.0850 ± 0.0008 ^b^	167.22 ± 2.54 ^b^	0.769 ± 0.014 ^e^	48.67 ± 1.15 ^e^	42.38 ± 0.94 ^e^
S19	0.540 ± 0.0099 ^f^	0.179 ± 0.0033 ^h^	1.016 ± 0.0124 ^cd^	2.21 ± 0.03 ^h^	0.0440 ± 0.0009 ^f^	171.12 ± 3.64 ^b^	0.415 ± 0.009 ^h^	82.33 ± 0.21 ^b^	38.81 ± 0.97 ^f^
C13	0.820 ± 0.0151 ^c^	0.200 ± 0.0037 ^fg^	1.018 ± 0.0125 ^cd^	3.78 ± 0.04 ^f^	0.0810 ± 0.0007 ^c^	176.60 ± 2.88 ^b^	0.560 ± 0.010 ^g^	50.33 ± 0.94 ^e^	77.62 ± 1.75 ^d^
C9	0.500 ± 0.0092 ^g^	0.211 ± 0.0039 ^fg^	0.959 ± 0.0117 ^e^	1.62 ± 0.02 ^i^	0.0440 ± 0.0009 ^f^	221.43 ± 3.34 ^a^	0.334 ± 0.008 ^i^	84.33 ± 1.93 ^a^	93.81 ± 1.23 ^a^
X9	0.760 ± 0.0140 ^d^	0.330 ± 0.0061 ^b^	0.996 ± 0.0122 ^d^	3.49 ± 0.04 ^g^	0.0850 ± 0.0009 ^b^	166.67 ± 2.85 ^b^	0.900 ± 0.016 ^d^	47.67 ± 0.72 ^e^	49.05 ± 0.14 ^e^
JK	0.490 ± 0.0090 ^g^	0.208 ± 0.0038 ^fg^	0.888 ± 0.0109 ^f^	0.67 ± 0.02 ^j^	0.0410 ± 0.0008 ^g^	295.61 ± 4.26 ^a^	0.441 ± 0.009 ^h^	86.70 ± 0.86 ^a^	85.04 ± 2.02 ^c^
Mean	0.635 ^B^	0.224 ^B^	0.979 ^B^	2.52 ^B^	0.0633 ^B^	199.78 ^A^	0.570 ^B^	66.67 ^A^	64.45 ^A^
LSD (*P* ≤ 0.05)									
Genotype (G)	0.031	0.011	0.029	0.12	0.0024	9.84	0.048	2.46	2.38
Salinity (S)	0.018	0.007	0.017	0.07	0.0014	5.68	0.028	1.42	1.37
G × S	0.044	0.016	0.041	0.17	0.0034	13.92	0.068	3.48	3.36

Values are means (n = 3). Mean values in each column followed by different lower-case letters were significantly different among genotypes, while different uppercase letters indicate significant differences among salinity treatments at P ≤ 0.05 according to Tukey’s HSD test

#### Chl b (mg g⁻¹ leaf fresh weight)

The Chl b content, an accessory pigment critical for light harvesting, also exhibited genotype-specific responses to salinity (Table 2). Under control conditions, Chl b ranged from 0.239 ± 0.004 mg g⁻¹ FW (S12) to 0.379 ± 0.007 mg g⁻¹ FW (X9). Salinity stress reduced these values to 0.179 ± 0.003–0.330 ± 0.006 mg g⁻¹ FW, with X9 again demonstrating superior retention (0.330 ± 0.006 mg g⁻¹ FW) and S19 exhibiting the lowest content (0.179 ± 0.003 mg g⁻¹ FW). The reduction in Chl b spanned 8.8% in S12 to 38.8% in C13, with pronounced decreases also observed in C9 (33.7%), JK (31.4%) and S19 (31.7%). The relatively mild reduction in S12, despite its low absolute values, suggests inherent differences in baseline pigment composition rather than stress tolerance.

#### TChl (mg g⁻¹ leaf fresh weight)

Consistent with trends observed for individual pigments, TChl content declined under salinity stress, with reductions ranging from 4.3% to 19.7% across genotypes (Table 2). Control values ranged from 1.041 ± 0.013 to 1.176 ± 0.014 mg g⁻¹ FW, with S12 and S19 exhibiting the highest values. Under saline conditions, TChl decreased to 0.888 ± 0.011–1.018 ± 0.013 mg g⁻¹ FW. C13 maintained the highest TChl under stress (1.018 ± 0.013 mg g⁻¹ FW), followed closely by S19 (1.016 ± 0.012 mg g⁻¹ FW), while JK showed the lowest content (0.888 ± 0.011 mg g⁻¹ FW). The relatively modest reduction in C13 (4.3%) indicates robust photosynthetic pigment retention under saline conditions, whereas the substantial declines in JK (19.7%), C9 (15.8%) and both S12, S19 hybrids (13.5–15.5%) suggest greater sensitivity. These findings highlight C13 and X9 as promising candidates for maintaining photosynthetic capacity under salinity stress. Analysis of variance indicated that genotype (G), salinity (S), and their interaction (G × S) had significant effects (*P* ≤ 0.05) on chlorophyll a, chlorophyll b, and total chlorophyll (Table 2; Fig. 5).

#### Gas exchange trait

Gas exchange traits were analyzed as an initial step in evaluating salinity tolerance among the genotypes. Variations in Gas Exchange traits among six citrus genotypes revealed significant difference in most traits on exposure to salt stress (Table 2; Fig. 5).

#### Leaf gas exchange parameters

##### A

Salinity stress significantly impaired photosynthetic gas exchange in citrus rootstocks, with the extent of inhibition varying significantly among genotypes (Table 2; Fig. 5). Under control conditions *A* ranged from 4.81 ± 0.03 µmol m⁻² s⁻¹ in JK to 7.69 ± 0.05 µmol m⁻² s⁻¹ in S19, while C13 also exhibited a high photosynthetic capacity (7.58 ± 0.05 µmol m⁻² s⁻¹). Exposure to salinity significantly reduced *A* across all genotypes, with values declining to 0.67 ± 0.02–3.78 ± 0.04 µmol m⁻² s⁻¹. Among the tested rootstocks, C13 maintained the highest photosynthetic rate under stress, closely followed by X9, whereas JK showed the most severe reduction. The percentage decline in *A* ranged from 44.3% in X9 to 86.1% in JK, highlighting the superior ability of X9 to sustain photosynthetic performance under saline conditions.

##### gₛ

Stomatal conductance (*gₛ*) exhibited a similar pattern of decline under salinity stress. Under control conditions, values were relatively uniform across genotypes, ranging from 0.0820 ± 0.0006 to 0.0900 ± 0.0011 mol m⁻² s⁻¹. Salinity exposure caused a reduction in gₛ to 0.0410 ± 0.0008–0.0850 ± 0.0009 mol m⁻² s⁻¹ (Table 2). Among the genotypes, S12 and X9 maintained the highest stomatal conductance under stress, whereas JK exhibited the greatest reduction, indicating pronounced stomatal closure. The percentage decline ranged from only 3.4% in S12 to 54.4% in JK, suggesting that tolerant genotypes were able to sustain stomatal function, while sensitive ones showed near-complete closure.

##### Ci

In contrast to the reductions observed in *A* and *gₛ*,* Ci* increased under salinity stress. Under control conditions, *Ci* ranged from 134.79 ± 3.07 to 158.58 ± 2.79 µmol mol⁻¹, but increased to 166.67 ± 2.85–295.61 ± 4.26 µmol mol⁻¹ under saline conditions (Table 2; Fig. 5). JK recorded the highest *Ci* (295.61 ± 4.26 µmol mol⁻¹; 119.3% increase), whereas X9 maintained the lowest values (166.67 ± 2.85 µmol mol⁻¹; 7.9% increase), closely followed by S12 (167.22 ± 2.54 µmol mol⁻¹). The increase in *Ci* in sensitive genotypes, despite reduced stomatal conductance, suggests the predominance of non-stomatal limitations.

##### E

*E* also declined substantially under stress (Table 2; Fig. 5). Control values ranged from 1.447 ± 0.018 to 1.970 ± 0.025 mmol H₂O m⁻² s⁻¹, with S19 exhibiting the highest transpiration. Salinity reduced E to 0.334 ± 0.008–0.900 ± 0.016 mmol H₂O m⁻² s⁻¹. Genotype X9 maintained the highest transpiration rate under stress (0.900 ± 0.016 mmol H₂O m⁻² s⁻¹), while C9 showed the most severe reduction (0.334 ± 0.008 mmol H₂O m⁻² s⁻¹). The percent decrease in *E* ranged from 41.1% in X9 to 78.9% in S19, consistent with the pattern observed for stomatal conductance.

Analysis of variance indicated significant effects of genotype (G), salinity (S), and their interaction (G × S) (*P* ≤ 0.05) on all gas-exchange parameters (Table 2). The significant G×S interaction highlights genotype-dependent variation in gas exchange responses under salinity.

### Reactive oxygen species and Lipid peroxidation

#### H₂O₂

Salinity stress triggered differential accumulation of reactive oxygen species and subsequent lipid peroxidation across citrus rootstocks, revealing marked variation in oxidative stress tolerance (Table 2; Fig. 5). Under control conditions, baseline H₂O₂ content ranged narrowly from 44.10 ± 0.45 to 47.60 ± 0.08 µmol g⁻¹ FW, with C9 (47.60 ± 0.08 µmol g⁻¹ FW) and JK (47.30 ± 1.11 µmol g⁻¹ FW) showing the highest values, while X9 (44.10 ± 0.45 µmol g⁻¹ FW) and S12 (44.20 ± 0.52 µmol g⁻¹ FW) maintained the lowest. Salinity exposure markedly elevated H₂O₂ levels across all genotypes, expanding the range to 47.67 ± 0.72–86.70 ± 0.86 µmol g⁻¹ FW. Genotype JK accumulated the highest H₂O₂ under stress (86.70 ± 0.86 µmol g⁻¹ FW), followed closely by C9 (84.33 ± 1.93 µmol g⁻¹ FW) and S19 (82.33 ± 0.21 µmol g⁻¹ FW). In striking contrast, X9 maintained the lowest H₂O₂ content under saline conditions (47.67 ± 0.72 µmol g⁻¹ FW), representing a mere 8.1% increase from control levels. The magnitude of H₂O₂ accumulation varied dramatically among genotypes, ranging from this modest 8.1% increase in X9 to an 83.3% surge in JK, with S19 (81.0%) and C9 (77.2%) also exhibiting substantial ROS accumulation.

#### MDA

Consistent with elevated ROS levels, MDA content (Table 2; Fig. 5) which is a reliable indicator of membrane lipid peroxidation, increased under salinity stress, though with considerable genotypic variation. Under non-saline conditions, MDA ranged from 24.22 ± 0.19 nmol g⁻¹ FW in S19 to 57.46 ± 0.79 nmol g⁻¹ FW in C13. Salinity stress elevated these values to 38.81 ± 0.97–93.81 ± 1.23 nmol g⁻¹ FW, with C9 exhibiting the highest lipid peroxidation (93.81 ± 1.23 nmol g⁻¹ FW), followed by JK (85.04 ± 2.02 nmol g⁻¹ FW) and C13 (77.62 ± 1.75 nmol g⁻¹ FW). Notably, S19 maintained the lowest MDA content under stress (38.81 ± 0.97 nmol g⁻¹ FW), suggesting superior membrane integrity despite its high H₂O₂ accumulation. The percent increase in MDA spanned from 31.9% in X9 to 86.6% in JK, with C9 (76.3%) and S19 (60.2%) also showing substantial membrane damage.

Two-way analysis of variance revealed significant effects of genotype (G), salinity level (S) and their interaction (G × S) on both H₂O₂ and MDA accumulation (*P* ≤ 0.05; Table 2). These findings underscore the genotype-specific nature of oxidative stress responses and highlight the differential capacity of the citrus rootstocks to mitigate salinity-induced oxidative damage.

### Osmolyte and metabolite accumulation

Salinity stress triggered a coordinated accumulation of osmolytes and secondary metabolites across citrus rootstocks, though the magnitude of response varied considerably among genotypes (Table 3; Fig. 5). The TSS, an indicator of soluble sugar accumulation, ranged from 11.88 ± 0.07 to 16.35 ± 0.21 mg g⁻¹ FW under control conditions, with X9 exhibiting the highest baseline value (16.35 ± 0.21 mg g⁻¹ FW) and S19 the lowest (11.88 ± 0.07 mg g⁻¹ FW). Exposure to salinity elevated TSS to 15.38 ± 0.22–23.45 ± 0.37 mg g⁻¹ FW, with X9 again maintaining the highest content (23.45 ± 0.37 mg g⁻¹ FW). The relative increase ranged from 25.2% in C9 to 43.4% in X9, suggesting enhanced osmotic adjustment capacity in the latter.

**Table 3:** Effect of salinity stress onosmolytes and metabolites: total soluble sugars (TSS) and proline (LPC) (µg g⁻¹ FW), total soluble proteins (TSP) (mg g⁻¹ FW) and total phenolics(TPh) (mg GAE g⁻¹ FW); antioxidant enzyme activities: SOD, APOX, CAT, GR and POX (U mg⁻¹ protein min⁻¹); in six citrus rootstocks

	TSS	LPC	TSP	TPh	SOD	APOX	CAT	GR	POX
Control									
S12	13.11 ± 0.15 ^f^	54.89 ± 1.40 ^bc^	7.87 ± 0.06 ^f^	7.78 ± 0.05 ^d^	8.29 ± 0.14 ^b^	7.19 ± 0.10 ^bc^	24.96 ± 0.55 ^c^	1.75 ± 0.02 ^f^	4.36 ± 0.09 ^a^
S19	11.88 ± 0.07 ^g^	57.19 ± 0.18 ^b^	8.11 ± 0.02 ^a^	6.82 ± 0.07 ^e^	7.69 ± 0.07 ^c^	6.49 ± 0.16 ^d^	26.03 ± 0.65 ^ab^	2.57 ± 0.01 ^a^	4.33 ± 0.08 ^a^
C13	14.67 ± 0.20 ^d^	42.35 ± 0.15 ^d^	7.36 ± 0.08 ^g^	8.27 ± 0.16 ^c^	7.18 ± 0.07 ^d^	6.88 ± 0.18 ^cd^	26.30 ± 0.38 ^a^	2.16 ± 0.02 ^c^	2.98 ± 0.01 ^d^
C9	13.38 ± 0.22 ^e^	33.08 ± 0.84 ^e^	7.86 ± 0.05 ^f^	9.73 ± 0.07 ^a^	9.40 ± 0.21 ^a^	7.02 ± 0.13 ^bc^	25.13 ± 0.21 ^bc^	1.89 ± 0.04 ^e^	2.85 ± 0.01 ^e^
X9	16.35 ± 0.21 ^a^	66.63 ± 1.23 ^a^	7.91 ± 0.04 ^e^	9.61 ± 0.07 ^b^	7.93 ± 0.04 ^bc^	8.11 ± 0.18 ^a^	25.13 ± 0.17 ^bc^	2.03 ± 0.00 ^d^	3.41 ± 0.07 ^c^
JK	14.48 ± 0.21 ^d^	54.57 ± 1.08 ^bc^	7.83 ± 0.14 ^f^	6.51 ± 0.11 ^f^	4.73 ± 0.08 ^e^	5.45 ± 0.06 ^e^	26.56 ± 0.44 ^a^	1.35 ± 0.00 ^g^	2.63 ± 0.01 ^f^
Mean	13.98 ^B^	51.45 ^B^	7.82 ^B^	8.12 ^B^	7.54 ^B^	6.86 ^B^	25.69 ^B^	1.96 ^B^	3.43 ^B^
Salinity									
S12	18.34 ± 0.41 ^e^	67.10 ± 1.48 ^d^	10.95 ± 0.19 ^a^	14.57 ± 0.29 ^b^	33.41 ± 0.41 ^b^	9.57 ± 0.07 ^b^	39.26 ± 0.77 ^d^	4.82 ± 0.03 ^d^	6.92 ± 0.01 ^c^
S19	15.38 ± 0.22 ^f^	66.42 ± 0.44 ^d^	9.17 ± 0.08 ^e^	9.66 ± 0.20 ^e^	24.06 ± 0.21 ^d^	7.87 ± 0.07 ^e^	37.23 ± 0.31 ^e^	5.73 ± 0.10 ^c^	7.79 ± 0.12 ^a^
C13	20.21 ± 0.22 ^c^	52.47 ± 0.78 ^e^	10.29 ± 0.01 ^c^	14.94 ± 0.05 ^b^	32.61 ± 0.84 ^bc^	8.99 ± 0.03 ^c^	35.83 ± 0.56 ^f^	6.23 ± 0.07 ^a^	4.40 ± 0.08 ^f^
C9	16.75 ± 0.17 ^e^	38.53 ± 0.21 ^f^	8.89 ± 0.10 ^f^	12.24 ± 0.11 ^d^	21.81 ± 0.35 ^e^	8.32 ± 0.20 ^d^	47.00 ± 0.34 ^b^	3.37 ± 0.02 ^e^	4.80 ± 0.05 ^e^
X9	23.45 ± 0.37 ^a^	84.34 ± 1.23 ^a^	11.38 ± 0.20 ^a^	17.59 ± 0.40 ^a^	36.63 ± 0.47 ^a^	11.44 ± 0.07 ^a^	31.16 ± 0.47 ^g^	5.84 ± 0.12 ^b^	4.80 ± 0.06 ^e^
JK	18.37 ± 0.23 ^d^	65.42 ± 1.09 ^d^	10.18 ± 0.02 ^c^	9.45 ± 0.11 ^e^	12.79 ± 0.10 ^f^	6.53 ± 0.12 ^f^	51.86 ± 0.87 ^a^	3.13 ± 0.07 ^f^	4.40 ± 0.07 ^f^
Mean	18.75 ^A^	62.38 ^A^	10.14 ^A^	13.07 ^A^	26.89 ^A^	8.79 ^A^	40.39 ^A^	4.85 ^A^	5.52 ^A^
LSD (*P* ≤ 0.05)									
Genotype (G)	0.72	4.11	0.29	0.36	0.6	0.46	1.68	0.09	0.26
Salinity (S)	0.42	2.37	0.17	0.21	0.34	0.27	0.97	0.05	0.15
G × S	1.01	5.81	0.41	0.51	0.84	0.66	2.38	0.13	0.37

Values are means (n = 3). Mean values in each column followed by different lower-case letters were significantly different among genotypes, while different uppercase letters indicate significant differences among salinity treatments at P ≤ 0.05 according to Tukey’s HSD test

LPC, a key compatible solute implicated in osmotic regulation and ROS detoxification, also accumulated differentially under stress (Table 3; Fig. 5). Control values ranged from 33.08 ± 0.84 µg g⁻¹ FW in C9 to 66.63 ± 1.23 µg g⁻¹ FW in X9. Under saline conditions, LPC content increased to 38.53 ± 0.21–84.34 ± 1.23 µg g⁻¹ FW, with percent increases spanning 16.1–26.6% across genotypes. Notably, X9 maintained the highest absolute LPC levels under both control and stress conditions, consistent with its superior osmotic adjustment capability.

Salinity induced marked increases in TSP, a parameter reflecting stress-responsive protein synthesis and broader metabolic reprogramming. Mean TSP values rose from a control range of 7.36 ± 0.08–8.11 ± 0.02 mg g⁻¹ FW to 8.89 ± 0.10–11.38 ± 0.20 mg g⁻¹ FW under salt stress. The greatest accumulation was observed in C13 (43.9% increase), with X9 showing a comparable response, indicative of robust metabolic adjustment under saline conditions.

As a measure of non-enzymatic antioxidant capacity, TPh displayed substantial genotypic variation in response to salinity. Under control conditions, values ranged from 6.51 ± 0.11 to 9.73 ± 0.07 mg GAE g⁻¹ FW. Following salt exposure, phenolic content increased to 9.45 ± 0.11–17.59 ± 0.40 mg GAE g⁻¹ FW, with X9 accumulating the highest levels and JK the lowest. Percent increases spanned from 25.8% in C9 to 87.3% in S12, suggesting genotype-specific activation of the phenylpropanoid pathway under saline stress. A significant Genotype × Salinity (G×S) interaction (*P* ≤ 0.05) indicated differential osmolyte and metabolite responses among the studied genotypes exposed to salinity stress (Table 3).

### Antioxidant enzyme activities

The enzymatic antioxidant system of citrus rootstocks responded to salinity stress with pronounced genotype-specific activation patterns (Table 3; Fig. 5). Under control conditions, SOD activity ranged from 4.73 ± 0.08 to 9.40 ± 0.21 units min⁻¹ mg⁻¹ protein, with C9 exhibiting the highest baseline activity and JK the lowest. Salinity exposure triggered a substantial increase in SOD activity across all genotypes, elevating values to 12.79 ± 0.10–36.63 ± 0.47 units min⁻¹ mg⁻¹ protein. X9 displayed the highest SOD activity under stress (36.63 ± 0.47 units min⁻¹ mg⁻¹ protein), followed closely by S19 (34.40 ± 0.52 units min⁻¹ mg⁻¹ protein), while JK maintained the lowest activity (12.79 ± 0.10 units min⁻¹ mg⁻¹ protein). The magnitude of SOD induction varied dramatically, ranging from 132.0% in C9 to 361.9% in S19, suggesting differential activation of superoxide-scavenging capacity.

APOX activity showed baseline values of 5.45 ± 0.06–8.11 ± 0.18 units min⁻¹ mg⁻¹ protein under control conditions. APOX activity is central to hydrogen peroxide detoxification. Salinity enhanced APOX activity to 6.53 ± 0.12–11.44 ± 0.07 units min⁻¹ mg⁻¹ protein, with X9 achieving the highest activity (11.44 ± 0.07 units min⁻¹ mg⁻¹ protein) and JK the lowest (6.53 ± 0.12 units min⁻¹ mg⁻¹ protein). The percent increase ranged from 18.5% in JK to 41.1% in X9, indicating genotype-dependent capacity for ascorbate-mediated H₂O₂ scavenging.

CAT activity under control conditions ranged narrowly from 24.96 ± 0.55 to 26.56 ± 0.44 units min⁻¹ mg⁻¹ protein. Salinity stress expanded this range to 31.16 ± 0.47–51.86 ± 0.87 units min⁻¹ mg⁻¹ protein, with JK unexpectedly exhibiting the highest activity (51.86 ± 0.87 units min⁻¹ mg⁻¹ protein) despite its susceptibility to oxidative damage. C13 also showed substantial CAT induction (47.00 ± 0.83 units min⁻¹ mg⁻¹ protein), while X9 maintained the lowest activity under stress (31.16 ± 0.47 units min⁻¹ mg⁻¹ protein). The percent increase ranged from 24.0% in X9 to 95.3% in JK.

GR is essential enzyme that maintains reduced glutathione pools, increased from control values of 1.35 ± 0.00–2.57 ± 0.01 units min⁻¹ mg⁻¹ protein to 3.13 ± 0.07–6.23 ± 0.07 units min⁻¹ mg⁻¹ protein under salinity. C13 displayed the highest GR activity under stress (6.23 ± 0.07 units min⁻¹ mg⁻¹ protein), followed by X9 (5.86 ± 0.09 units min⁻¹ mg⁻¹ protein). The percent increase ranged from 78.3% in JK to 188.4% in C13.

POX activity under control conditions ranged from 2.63 ± 0.01 to 4.36 ± 0.09 units min⁻¹ mg⁻¹ protein, increasing to 4.40 ± 0.07–7.79 ± 0.12 units min⁻¹ mg⁻¹ protein under salinity. S19 exhibited the highest POX activity under stress (7.79 ± 0.12 units min⁻¹ mg⁻¹ protein), followed by S12 (6.92 ± 0.10 units min⁻¹ mg⁻¹ protein). The percent increase spanned 40.8% to 79.9% across genotypes.

Analysis of variance confirmed significant effects of genotype (G), salinity (S) and their interaction (G × S) on all antioxidant enzyme activities (*P* ≤ 0.05) under the NaCl-induced salinity stress.

### Leaf mineral ion composition

Salinity stress profoundly disrupted mineral ion homeostasis in citrus rootstocks, with genotypes exhibiting markedly different capacities for maintaining essential nutrient uptake while restricting toxic ion accumulation (Table 4; Fig. 5). Ions like K⁺, a critical osmotic regulator and enzyme cofactor, declined substantially under saline conditions. Control values ranged from 17.2 ± 0.1 mg g⁻¹ DW in C13 to 23.4 ± 0.3 mg g⁻¹ DW in X9. Salinity exposure reduced K⁺ content to 9.5 ± 0.1–16.9 ± 0.3 mg g⁻¹ DW, with X9 maintaining the highest levels (16.9 ± 0.3 mg g⁻¹ DW) and C9 the lowest (9.5 ± 0.1 mg g⁻¹ DW). The magnitude of K⁺ reduction varied widely, ranging from 27.8% in X9 to 56.3% in S19, highlighting the superior K⁺ retention capacity of X9 under salt stress.

**Table 4:** Effect of salinity stress on leaf and root mineral ion composition: K⁺, Ca²⁺, Na⁺, Cl⁻ and Mg²⁺ (mg g⁻¹ DW); and chloroplast ultrastructural traits: chloroplast area (CA) (µm²) and starch grain (SG) number in six citrus rootstocks

	LK+	LCa2+	LNa+	LCl-	LMg2+	RK+	RCa2+	RNa+	RCl-	RMg2+	CA	SG
Control												
S12	2.03 ± 0.03 ^d^	4.29 ± 0.07 ^d^	0.35 ± 0.00 ^b^	0.18 ± 0.00 ^a^	0.91 ± 0.02 ^c^	1.70 ± 0.012 ^b^	3.67 ± 0.006 ^b^	0.219 ± 0.008 ^h^	0.166 ± 0.005 ^i^	0.44 ± 0.003^cb^	8.4 ± 0.5^bc^	3.3 ± 0.3^a^
S19	2.31 ± 0.03 ^a^	5.50 ± 0.05 ^a^	0.25 ± 0.00 ^d^	0.16 ± 0.00 ^c^	0.90 ± 0.01 ^cd^	1.57 ± 0.006 c	3.69 ± 0.047 ^b^	0.248 ± 0.009 ^h^	0.163 ± 0.005^i^	0.43 ± 0.003 ^c^	8.3 ± 0.5^c^	3.2 ± 0.3^a^
C13	1.72 ± 0.01 ^f^	2.35 ± 0.02 ^g^	0.24 ± 0.00 ^e^	0.17 ± 0.00 ^b^	0.83 ± 0.02 ^e^	1.33 ± 0.024 ^e^	2.10 ± 0.032 ^g^	0.333 ± 0.012 ^g^	0.181 ± 0.006^i^	0.41 ± 0.003 ^cd^	8.7 ± 0.5^a^	3.2 ± 0.3^a^
C9	1.79 ± 0.03 ^e^	4.36 ± 0.10 ^c^	0.34 ± 0.00 ^c^	0.16 ± 0.00 ^c^	0.91 ± 0.00 ^c^	1.41 ± 0.014 ^d^	2.03 ± 0.033 ^g^	0.340 ± 0.014 ^g^	0.165 ± 0.005 ^i^	0.42 ± 0.003 ^c^	8.4 ± 0.5^bc^	3.3 ± 0.3^a^
X9	2.34 ± 0.03 ^a^	4.04 ± 0.04 ^e^	0.23 ± 0.00 ^f^	0.18 ± 0.00 ^a^	1.05 ± 0.02 ^a^	1.84 ± 0.012 ^a^	3.81 ± 0.026 ^a^	0.229 ± 0.006 ^h^	0.178 ± 0.006 ^i^	0.46 ± 0.000 ^ab^	8.5 ± 0.5^ab^	3.3 ± 0.3^a^
JK	2.25 ± 0.06 ^b^	2.44 ± 0.03 ^f^	0.36 ± 0.00 ^a^	0.15 ± 0.00 ^d^	0.87 ± 0.00 ^d^	1.68 ± 0.042 ^b^	2.11 ± 0.047 ^g^	0.347 ± 0.011 ^g^	0.151 ± 0.004^i^	0.42 ± 0.012 ^c^	8.4 ± 0.6^bc^	3.4 ± 0.4^a^
Mean	2.07 ^A^	3.83 ^A^	0.30 ^B^	0.17 ^B^	0.91 ^A^	1.59 ^A^	2.90 ^A^	0.286 ^B^	0.167 ^B^	0.43 ^A^	8.45^B^	3.28^A^
Salinity												
S12	1.36 ± 0.02 ^b^	3.31 ± 0.07 ^b^	0.73 ± 0.01 ^e^	0.96 ± 0.02 ^e^	0.80 ± 0.00 ^b^	1.08 ± 0.006 ^b^	2.87 ± 0.026 ^e^	0.694 ± 0.015 ^d^	0.801 ± 0.014 ^f^	0.37 ± 0.006 ^d^	8.8 ± 0.5^d^	2.3 ± 0.3^b^
S19	1.01 ± 0.02 ^d^	4.07 ± 0.00 ^a^	1.54 ± 0.04 ^b^	1.52 ± 0.00 ^d^	0.48 ± 0.01 ^e^	0.90 ± 0.003 ^d^	2.73 ± 0.006 e	1.564 ± 0.028 ^b^	1.528 ± 0.026 ^c^	0.24 ± 0.003 ^e^	10.1 ± 0.7^b^	1.2 ± 0.2^d^
C13	1.20 ± 0.00 ^c^	1.75 ± 0.00 ^e^	0.68 ± 0.01 ^f^	0.82 ± 0.02 ^f^	0.74 ± 0.02 ^c^	0.96 ± 0.021 ^c^	1.61 ± 0.010 ^h^	0.725 ± 0.018 ^d^	0.958 ± 0.015 ^e^	0.35 ± 0.007 ^d^	9.3 ± 0.5^c^	1.9 ± 0.3^c^
C9	0.95 ± 0.01 ^e^	3.01 ± 0.01 ^c^	0.95 ± 0.00 ^d^	2.37 ± 0.01 ^b^	0.65 ± 0.01 ^d^	0.85 ± 0.010 ^e^	1.48 ± 0.021 ^h^	0.939 ± 0.022 ^c^	2.303 ± 0.038 ^b^	0.32 ± 0.000 ^d^	10.8 ± 0.8^a^	0.9 ± 0 .2^e^
X9	1.69 ± 0.03 ^a^	3.24 ± 0.00 ^b^	0.38 ± 0.01 ^g^	0.78 ± 0.00 ^f^	0.95 ± 0.01 ^a^	1.30 ± 0.009 ^a^	3.18 ± 0.054 ^d^	0.382 ± 0.011 ^f^	0.739 ± 0.012 ^f^	0.43 ± 0.003 ^cb^	8.9 ± 0.4^cd^	2.4 ± 0.3^b^
JK	1.11 ± 0.01 ^d^	1.42 ± 0.01 ^f^	1.38 ± 0.02 ^c^	2.61 ± 0.00 ^a^	0.48 ± 0.00 ^e^	0.90 ± 0.010 ^d^	1.19 ± 0.026 ^i^	1.336 ± 0.024 ^b^	2.613 ± 0.042 ^a^	0.24 ± 0.000 ^e^	11.2 ± 0.9^a^	0.8 ± 0.2^e^
Mean	1.22 ^B^	2.80 ^B^	0.94 ^A^	1.51 ^A^	0.68 ^B^	1.01 ^B^	2.18 ^B^	0.940 ^A^	1.490 ^A^	0.33 ^B^	9.85^A^	1.58^B^
LSD (*P* ≤ 0.05)												
Genotype (G)	0.1	0.27	0.01	0.01	0.05	0.048	0.27	0.048	0.064	0.05	1.2	0.5
Salinity (S)	0.06	0.16	0.01	0.01	0.03	0.028	0.16	0.028	0.037	0.03	0.7	0.3
G × S	0.14	0.38	0.02	0.02	0.07	0.068	0.38	0.068	0.09	0.07	1.7	0.7

Values are means (n = 3). Mean values in each column followed by different lower-case letters were significantly different among genotypes, while different uppercase letters indicate significant differences among salinity treatments at P ≤ 0.05 according to Tukey’s HSD test

Salinity stress reduced leaf calcium (LCa²⁺) content across all genotypes (Table 4; Fig. 5). Under control conditions, values ranged from 2.35 ± 0.02 to 5.50 ± 0.05 mg g⁻¹ DW, with S19 showing the highest and C13 the lowest levels. Under salinity, LCa²⁺ declined to 1.42 ± 0.01–4.07 ± 0.00 mg g⁻¹ DW, with S19 maintaining the highest content and JK the lowest. The mean LCa²⁺ decreased from 3.83 to 2.80 mg g⁻¹ DW under stress.

Leaf Mg²⁺, central to chlorophyll structure and photosynthetic function, decreased from a control range of 8.3 ± 0.2–10.5 ± 0.2 mg g⁻¹ DW to 4.8 ± 0.1–9.5 ± 0.1 mg g⁻¹ DW under salinity. X9 demonstrated remarkable Mg²⁺ retention with only a 9.5% reduction, maintaining the highest content under stress (9.5 ± 0.1 mg g⁻¹ DW). In contrast, S19 suffered the most severe Mg²⁺ decline (46.7%), consistent with its pronounced chlorophyll degradation and photosynthetic impairment.

Conversely, toxic ion accumulation escalated dramatically under saline conditions. Leaf Na⁺ content increased from baseline levels of 2.3 ± 0.0–3.6 ± 0.0 mg g⁻¹ DW to 3.8 ± 0.1–15.4 ± 0.4 mg g⁻¹ DW under salinity. X9 exhibited the most effective Na⁺ exclusion, accumulating only 3.8 ± 0.1 mg g⁻¹ DW (65.2% increase) while S19 accumulated the highest Na⁺ load (15.4 ± 0.4 mg g⁻¹ DW), representing a staggering 516.0% increase. JK and C9 also showed substantial Na⁺ accumulation (13.8 ± 0.3 and 12.4 ± 0.3 mg g⁻¹ DW, respectively), correlating with their pronounced oxidative damage and growth suppression.

Leaf Cl⁻ accumulation followed a similar pattern, with control values of 1.5 ± 0.0–1.8 ± 0.0 mg g⁻¹ DW surging to 7.8 ± 0.0–26.1 ± 0.0 mg g⁻¹ DW under salinity. X9 again demonstrated superior Cl⁻ exclusion with the lowest accumulation (7.8 ± 0.0 mg g⁻¹ DW; 333% increase), while JK accumulated the highest Cl⁻ levels (26.1 ± 0.0 mg g⁻¹ DW; 1,640% increase), followed by C9 (23.7 ± 0.0 mg g⁻¹ DW; 1,381% increase).

Two-way ANOVA indicated significant effects of genotype (G), salinity (S) and their interaction (G × S) on all measured mineral ions (*P* ≤ 0.05; Table 4). X9 exhibited superior ion homeostasis under stress, maintaining high K⁺, Ca²⁺ and Mg²⁺ levels while effectively excluding Na⁺ and Cl⁻. This maintenance of favourable K⁺/Na⁺ and Ca²⁺/Na⁺ ratios likely underpins its sustained photosynthesis, reduced oxidative damage and overall salt tolerance.

### Root mineral ion composition

A comparison of RK⁺ concentrations among the genotypes revealed that salinity induced by NaCl significantly reduced RK⁺ accumulation, with clear genotype-specific retention patterns (Table 4; Fig. 5). Under control conditions, RK⁺ content ranged from 1.33 ± 0.024 mg g⁻¹ DW in C13 to 1.84 ± 0.012 mg g⁻¹ DW in X9. Following salt exposure, these values declined to 0.85 ± 0.010–1.30 ± 0.009 mg g⁻¹ DW. X9 retained the highest K⁺ level under stress (1.30 ± 0.009 mg g⁻¹ DW; 29.3% decrease), whereas C9 showed the lowest (0.85 ± 0.010 mg g⁻¹ DW; 39.7% reduction). S19 and JK also exhibited substantial K⁺ depletion (0.90 ± 0.003 and 0.90 ± 0.010 mg g⁻¹ DW, respectively), representing decreases of 42.7% and 46.4%.

NaCl stress also significantly affected root Ca²⁺ accumulation, with clear genotype-specific patterns emerging (Table 4). The Ca²⁺ content ranged from 2.03 ± 0.033 mg g⁻¹ DW in C9 to 3.81 ± 0.026 mg g⁻¹ DW in X9 under control conditions. Exposure to salinity stress reduced these concentrations to between 1.19 ± 0.026 and 3.18 ± 0.054 mg g⁻¹ DW. X9 again retained the highest Ca²⁺ level under stress (3.18 ± 0.054 mg g⁻¹ DW; a 16.5% decrease), whereas JK recorded the lowest value (1.19 ± 0.026 mg g⁻¹ DW; a 43.6% reduction). Genotype C9 also showed considerable Ca²⁺ depletion, falling to 1.48 ± 0.021 mg g⁻¹ DW (a 27.1% decrease).

Examination of root ion concentrations revealed that salinity stress dramatically altered sodium and chloride accumulation patterns, with genotype-specific responses becoming readily apparent (Table 4). Root Na⁺ content under control conditions ranged from 2.19 ± 0.08 mg g⁻¹ DW in S12 to 3.47 ± 0.11 mg g⁻¹ DW in JK. Following salt exposure, these values expanded to 3.82 ± 0.11–15.64 ± 0.28 mg g⁻¹ DW. X9 exhibited the lowest Na⁺ accumulation under stress (3.82 ± 0.11 mg g⁻¹ DW; 66.8% increase), while S19 accumulated the highest (15.64 ± 0.28 mg g⁻¹ DW; 530.7% increase). JK and C9 also showed substantial Na⁺ loading (14.28 ± 0.25 and 13.45 ± 0.22 mg g⁻¹ DW, respectively), with increases of 408.2% and 395.6%.

Root Cl⁻ content followed a comparable trend. Control values ranged from 1.51 ± 0.04 to 1.81 ± 0.06 mg g⁻¹ DW across genotypes. Under salinity, Cl⁻ concentrations rose sharply to 7.39 ± 0.12–26.13 ± 0.42 mg g⁻¹ DW. X9 maintained the lowest Cl⁻ accumulation (7.39 ± 0.12 mg g⁻¹ DW; 315.2% increase), whereas JK accumulated the highest (26.13 ± 0.42 mg g⁻¹ DW; 1,630.5% increase). C9 and S19 also displayed pronounced Cl⁻ loading (23.45 ± 0.38 and 21.67 ± 0.35 mg g⁻¹ DW, respectively), representing increases of 1,381.2% and 1,334.8%.

Salinity exposure significantly reduced RMg²⁺ accumulation, and this effect varied considerably across genotypes (Table 4). Under control conditions, Mg²⁺ levels ranged from 0.41 ± 0.003 mg g⁻¹ DW in C13 to 0.46 ± 0.000 mg g⁻¹ DW in X9. After NaCl treatment the values declined to between 0.24 ± 0.003 and 0.43 ± 0.003 mg g⁻¹ DW. X9 maintained the highest Mg²⁺ content under stress (0.43 ± 0.003 mg g⁻¹ DW), declining by only 6.5% relative to its control. In contrast, S19 and JK showed the lowest levels (0.24 ± 0.003 and 0.24 ± 0.000 mg g⁻¹ DW, respectively), with reductions of 44.2% and 42.9%. Notably, S12 also retained Mg²⁺ relatively well (0.37 ± 0.006 mg g⁻¹ DW; a 15.9% decrease), as did C9 (0.32 ± 0.000 mg g⁻¹ DW; a 23.8% decrease). Analysis of variance indicated significant effects of genotype (G), salinity (S), and their interaction (G × S) (*P* ≤ 0.05) on all root ion traits (K⁺, Ca²⁺, Mg²⁺, Na⁺, and Cl⁻) (Table 4).

### Integrated multivariate analysis

#### Pearson correlation analysis

**Fig. 1:**
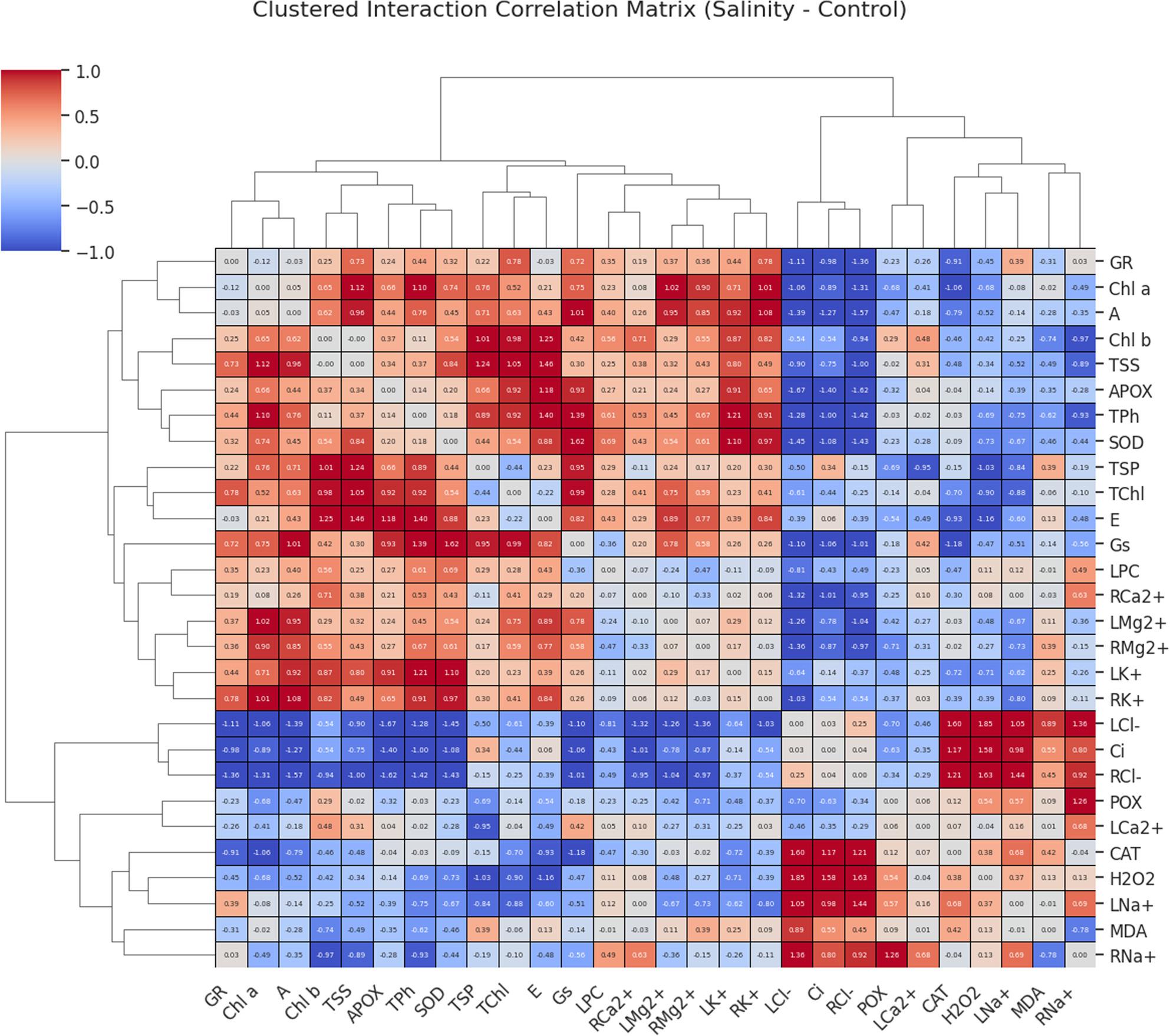
Pearson correlation heatmap of physiological, biochemical and mineral parameters in citrus rootstocks under salinity stress. The matrix displays Pearson correlation coefficients (r) among all measured traits across six citrus rootstocks under salinity stress. The color gradient represents the strength and direction of correlations, ranging from deep blue (negative correlation, -1.0) to deep red (positive correlation, + 1.0). Hierarchical clustering (dendrograms) on the top and left axes groups parameters with similar correlation patterns. Abbreviations and units: Gas Exchange: Net photosynthetic rate (A, µmol m⁻² s⁻¹); Stomatal conductance (gₛ, mol m⁻² s⁻¹); Intercellular CO₂ concentration (C_i_, µmol mol⁻¹); Transpiration rate (E, mmol H₂O m⁻² s⁻¹). Osmolytes & Metabolites: Total soluble sugars (TSS, mg g⁻¹ FW); Proline (LPC, µg g⁻¹ FW); Total soluble proteins (TSP, mg g⁻¹ FW); Total phenolics (TPh, mg GAE g⁻¹ FW). Oxidative Stress & Antioxidants: Hydrogen peroxide (H₂O₂, µmol g⁻¹ FW); Malondialdehyde (MDA, nmol g⁻¹ FW); Superoxide dismutase (SOD), Ascorbate peroxidase (APOX), Catalase (CAT), Glutathione reductase (GR) and Peroxidase (POX) (U mg⁻¹ protein min⁻¹). Mineral Composition: Leaf ions (L followed by element, e.g., LK⁺, LCa²⁺, LMg²⁺, LNa⁺, LCl⁻) and Root ions (R followed by element, e.g., RK⁺, RCa²⁺, RNa⁺, RCl⁻, RMg²⁺), all measured in mg g⁻¹ DW

Pearson correlation analysis, visualized through a heatmap with hierarchical clustering (Fig. 1), revealed two distinct physiological and biochemical clusters under salinity stress, with the first representing a coordinated adaptive module of growth, metabolism, and defense—featuring strong positive correlations among photosynthetic parameters (*A*,* g*_*s*_, *E*, chlorophyll pigments), antioxidant and osmotic components (TPh, APOX, SOD, TSS, TSP), and essential nutrients (root and leaf K⁺, Mg²⁺), all linked to maintained growth—while the second cluster grouped traits of ion toxicity and oxidative damage, where toxic ions (LCl⁻, RCl⁻, LNa⁺, RNa⁺) correlated positively with each other and with *Ci*, and oxidative stress markers (H₂O₂, MDA) clustered closely CAT activity, suggesting CAT acts as a reactive response to existing damage rather than a preventive defense. Importantly, the two clusters showed a dominant negative correlation (deep blue zones), where Na⁺ and Cl⁻ accumulation inversely related to growth rate, chlorophyll content, and gas exchange, confirming that ion toxicity is the primary driver of physiological decline in sensitive genotypes, suppressing the adaptive module and triggering oxidative damage.

#### Hierarchical clustering

**Fig. 2:**
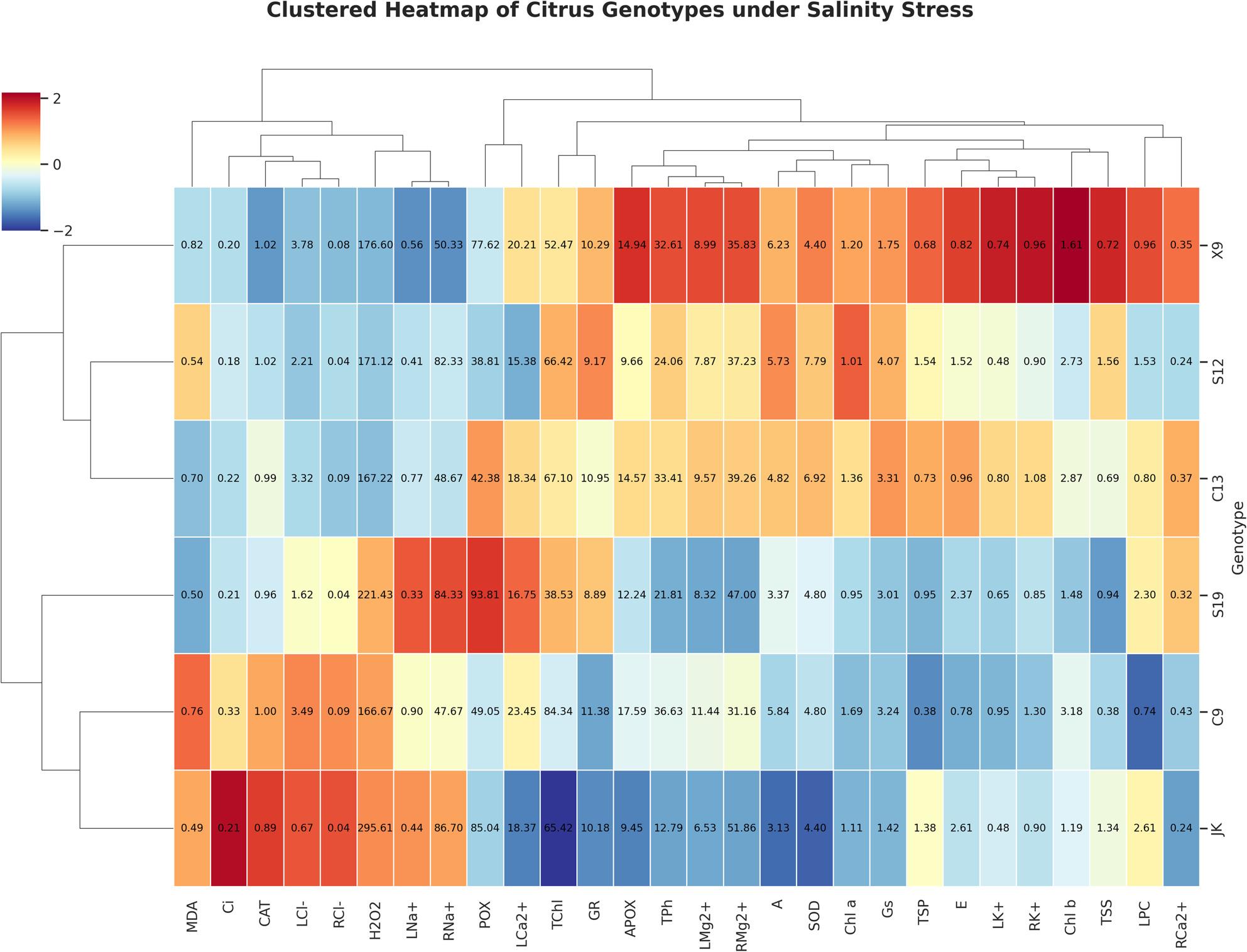
The heatmap represents the standardized response (z-scores) of various citrus genotypes to salinity stress. The color intensity indicates the level of expression or concentration: Red (positive z-scores) represents higher values relative to the mean, while Blue (negative z-scores) represents lower values. Hierarchical clustering on the Y-axis groups genotypes based on their overall response similarity and clustering on the X-axis groups parameters that show similar trends across genotypes. Genotypes include X-639 and Jatti Khatti, SCSH 17 − 12 and SCSH 9–19 and CRH 21 − 13 and CRH 21 − 9. Parameter abbreviations: Gas Exchange: Net photosynthesis (A), Stomatal conductance (Gs), Intercellular CO₂ concentration (Ci) and Transpiration rate (E). Oxidative Stress & Defense: Malondialdehyde (MDA), Hydrogen peroxide (H₂O₂), Catalase (CAT), Peroxidase (POX), Glutathione reductase (GR), Ascorbate peroxidase (APOX), Superoxide dismutase (SOD) and Total phenolics (TPh). Osmolytes & Nutrition: Proline (LPC), Total soluble sugars (TSS), Total soluble proteins (TSP) and leaf and root mineral ions (LCl⁻, LNa⁺, LCa²⁺, LMg²⁺, LK⁺, RK⁺, RCa²⁺, RNa⁺, RCl⁻, RMg²⁺)

Hierarchical clustering (Fig. 2) of both genotypes and traits under salinity stress clearly separated the citrus genotypes into two distinct groups: a tolerant cluster (X9, S12, C13) characterized by a “performance-oriented” profile, with X9 being the most resilient and showing strong positive responses in gas exchange (*A*,* g*_*s*_, *E*), chlorophyll content (Chl a, Chl b), and enzymatic antioxidants (SOD, APOX); and a sensitive cluster (JK, C9, S19) displaying a “stress-susceptible” profile, marked by high accumulation of toxic ions (LCl⁻, RCl⁻, LNa⁺, RNa⁺) and oxidative stress markers (MDA, H₂O₂). Concurrently, trait clustering partitioned the measured variables into two functional modules: a toxicity and reactive module grouping MDA, *Ci*, CAT, and toxic ions, where elevated *Ci* and CAT activity in sensitive genotypes (JK, C9) reflect physiological distress and reactive responses rather than effective defense; and an adaptive performance module integrating growth (GR), gas exchange (*A*,* gs*,* E*), osmolytes (TSS, TSP), and essential nutrients (LK⁺, RK⁺, LMg²⁺, RMg²⁺), where tolerant genotypes (especially X9) maintained high levels, indicating robust osmotic adjustment and nutrient homeostasis supporting sustained photosynthesis. Overall, these clustering results demonstrate that tolerance in genotypes like X9 arises from the simultaneous maintenance of nutrient balance and gas exchange, whereas sensitivity in genotypes like JK stems from a failure in ion exclusion, leading to substantial lipid peroxidation and metabolic disruption.

#### PCA

**Fig. 3:**
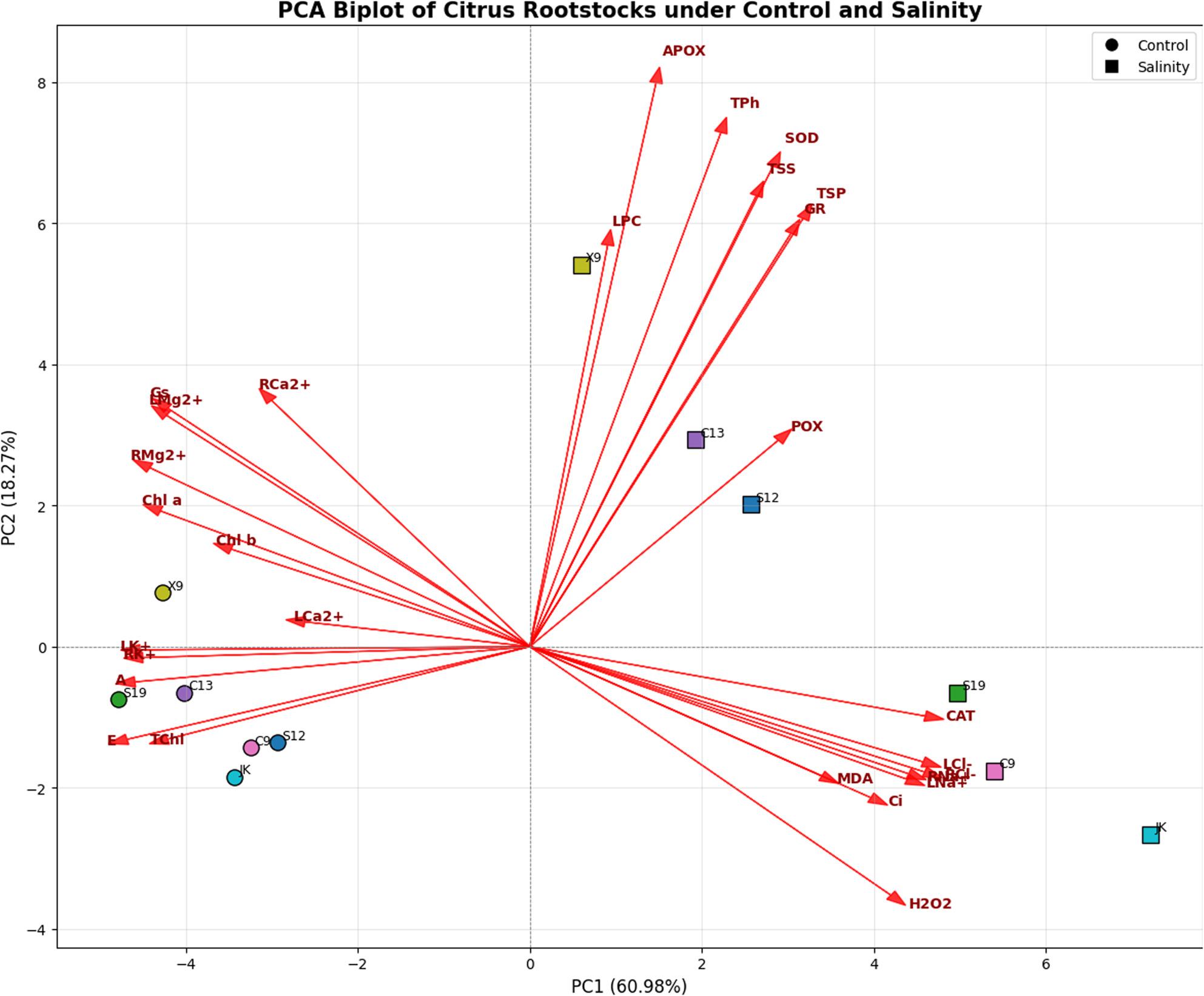
Principal Component Analysis (PCA) biplot showing the relationships among physiological, biochemical, antioxidant, and mineral ion traits in six citrus rootstocks under control and salinity stress conditions. The first two principal components explained 79.25% of the total variance (PC1: 60.98%; PC2: 18.27%). Circles (●) represent control and squares (■) represent salinity-treated plants. PC1 primarily separated control samples (negative axis), associated with photosynthesis (A, E, gs) and essential nutrients (LK⁺, RK⁺, RMg²⁺), from salinity-stressed samples (positive axis), associated with toxic ions (LNa⁺, LCl⁻, RCl⁻) and oxidative stress markers (MDA, H₂O₂). PC2 distinguished genotypic responses, with tolerant genotypes (e.g., X9) associated with antioxidant enzymes (SOD, APOX) and osmolytes (TSS, TSP, TPh), while sensitive genotypes (e.g., JK, C9) aligned with Ci and H₂O₂

The PCA effectively visualized (Fig. 3) the multifaceted relationships between genotypes, treatments, and physiological traits, with the first principal component (PC1, explaining 60.98% of variance) serving as the primary axis of salinity impact: all control samples clustered on the negative side of PC1, strongly associated with photosynthesis (*A*,* E*,* g*_*s*_) and essential nutrients (LK⁺, RK⁺, RMg²⁺), confirming high metabolic vigor under non-stress conditions, while salt-stressed plants shifted toward the positive PC1 axis driven by toxic ion accumulation (LNa⁺, LCl⁻, RCl⁻) and oxidative damage markers (MDA, H₂O₂). The second principal component (PC2, 18.27%) further differentiated genotypic adaptive strategies under stress: the tolerant response, exemplified by genotype X9 uniquely positioned in the upper-right quadrant, showed a powerful association with antioxidant defense (APOX, SOD) and osmotic regulators (TSS, TSP, TPh), with long vectors for APOX and growth rate (GR) indicating that its tolerance derives from highly efficient ROS scavenging and sustained biomass accumulation; in contrast, sensitive genotypes JK and C9 occupied the lower-right quadrant, aligning closely with *Ci* and H₂O₂ vectors, pointing to non-stomatal photosynthetic limitations and a lack of effective antioxidant mitigation. The biplot also revealed a strong negative correlation (nearly 180° vector orientation) between growth-related traits on the left and ion toxicity markers on the right, with the proximity of *Ci* to toxic ion vectors suggesting that as Na⁺ and Cl⁻ levels rise, plants lose the ability to fix CO₂, leading to its accumulation in sub-stomatal cavities.

#### Genotype ranking

**Fig. 4:**
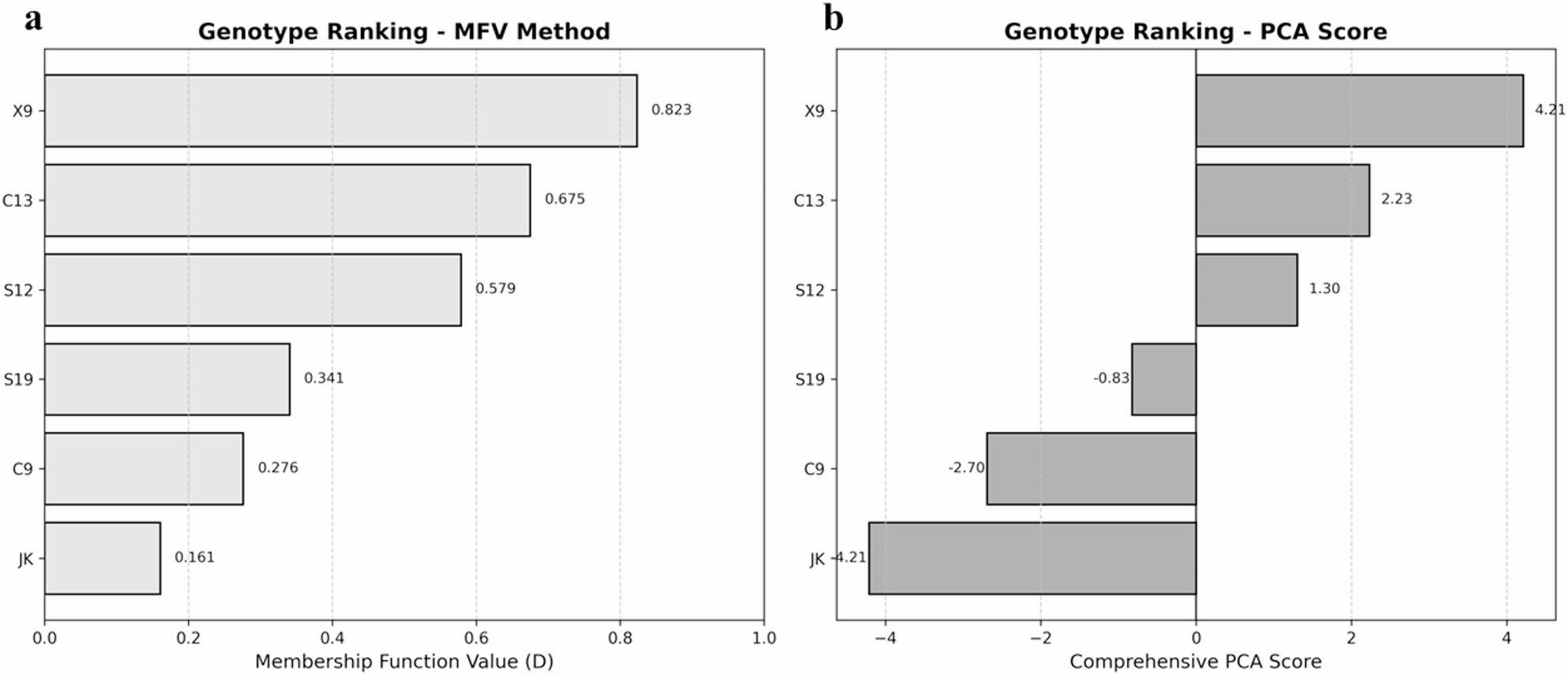
Comparative ranking of citrus rootstocks for salt tolerance based on (**a**) Membership Function Value (MFV) scores and (**b**) Principal Component Analysis (PCA) composite scores. The bar charts represent the relative salt tolerance of six citrus genotypes. High positive scores in both models indicate superior tolerance, while lower or negative scores indicate sensitivity. (**a**) MFV Ranking: Represents the mean membership function value across all physiological and biochemical parameters. Scores range from 0 to 1, where higher values reflect a comprehensive "balanced" response to stress. (**b**) PCA Ranking: Represents the composite score derived from the primary principal components. This ranking emphasizes the traits that contribute most significantly to the total variance observed in the study. Genotypic Order: Under the present experimental conditions, both models consistently indicated the following descending order of salinity tolerance: X-639 > CRH 21-13 > SCSH 17-12 > SCSH 9-19 > CRH 21-9 > Jatti Khatti. Statistical Significance: The strong agreement between the two independent mathematical models (MFV and PCA) supports the reliability of the tolerance screening process under the tested experimental conditions

**Fig. 5:**
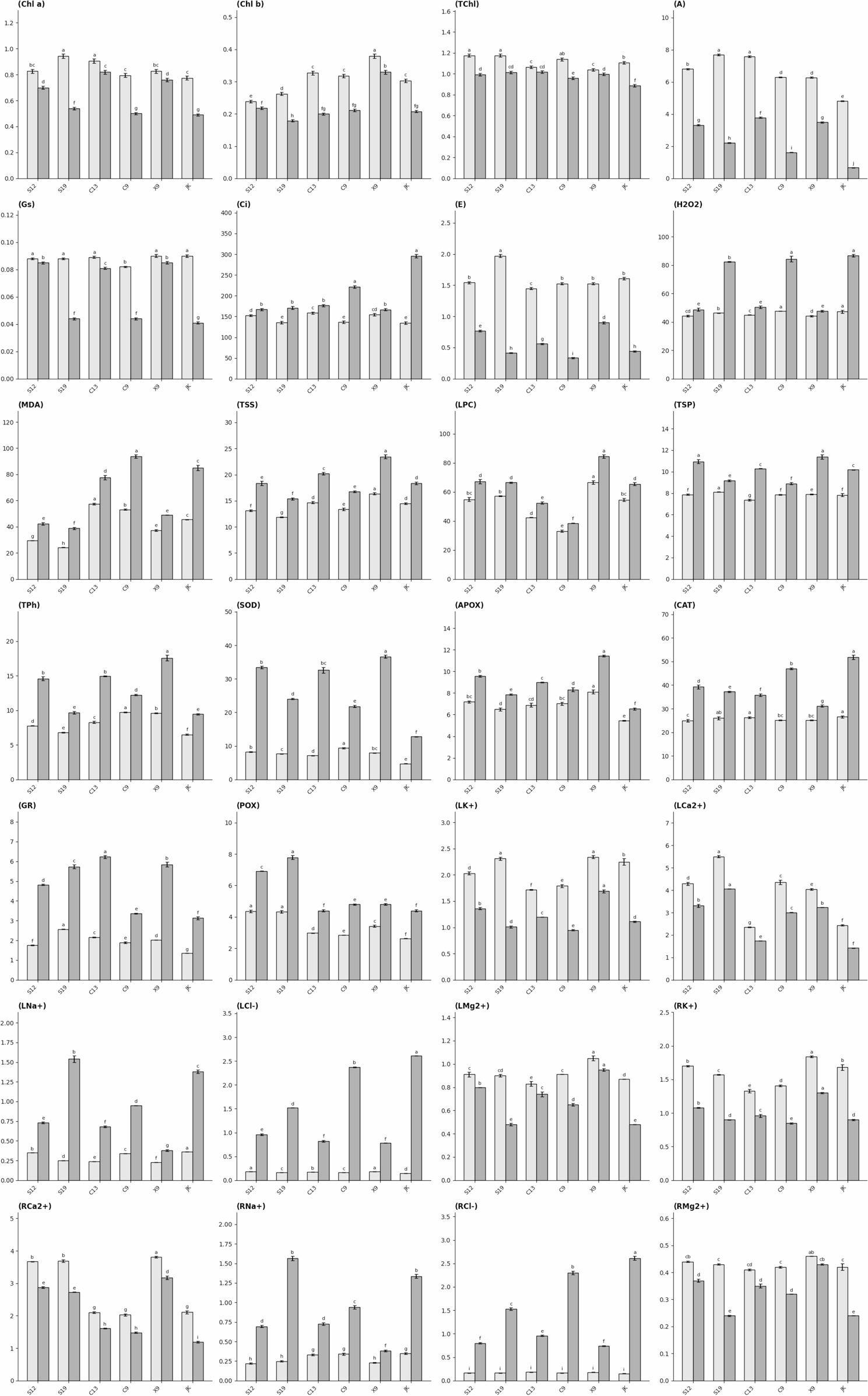
Bar charts showing the response of six citrus rootstocks (CRH 21 − 13, CRH 21 − 9, Jatti Khatti, SCSH 17 − 12, SCSH 9–19 and X-639) to salinity stress across all measured physiological, biochemical and mineral parameters. Light grey bars represent Control (0 mM NaCl) conditions and dark grey bars represent Salinity stress. Data are presented as mean ± standard error (SE). Different lowercase letters above the bars indicate significant differences (*P* < 0.05) among the treatments and genotypes based on Tukey’s HSD test. Genotypes like X-639 show better maintenance of essential nutrients (LK⁺, LCa²⁺) and lower accumulation of toxic ions (LNa⁺, LCl⁻) compared to sensitive types like Jatti Khatti. Abbreviations and Units: Gas Exchange Parameters: A (Net photosynthetic rate, µmol m⁻² s⁻¹), Gs (Stomatal conductance, mol m⁻² s⁻¹), Ci (Intercellular CO₂ concentration, µmol mol⁻¹), E (Transpiration rate, mmol H₂O m⁻² s⁻¹). Photosynthetic Pigments: Chl a (Chlorophyll a, mg g⁻¹ FW), Chl b (Chlorophyll b, mg g⁻¹ FW), TChl (Total chlorophyll, mg g⁻¹ FW). Oxidative Stress & Antioxidant Enzymes: H₂O₂ (Hydrogen peroxide, µmol g⁻¹ FW), MDA (Malondialdehyde, nmol g⁻¹ FW), SOD (Superoxide dismutase, U mg⁻¹ protein), APOX (Ascorbate peroxidase, U mg⁻¹ protein), CAT (Catalase, U mg⁻¹ protein), GR (Glutathione reductase, U mg⁻¹ protein), POX (Peroxidase, U mg⁻¹ protein). Osmolytes & Secondary Metabolites: TSS (Total soluble sugars, mg g⁻¹ FW), LPC (Leaf proline content, µg g⁻¹ FW), TSP (Total soluble proteins, mg g⁻¹ FW), TPh (Total phenolics, mg GAE g⁻¹ FW). Mineral Ion Composition: LK⁺ (Leaf potassium, mg g⁻¹ DW), LCa²⁺ (Leaf calcium, mg g⁻¹ DW), LMg²⁺ (Leaf magnesium, mg g⁻¹ DW), LNa⁺ (Leaf sodium, mg g⁻¹ DW), LCl⁻ (Leaf chloride, mg g⁻¹ DW), RK⁺(Root potassium, mg g⁻¹ DW), RCa²⁺(Root calcium, mg g⁻¹ DW), RMg²⁺(Root magnesium, mg g⁻¹ DW), RNa⁺ (Root sodium, mg g⁻¹ DW), RCl⁻ (Root chloride, mg g⁻¹ DW)

To provide a robust evaluation of salinity tolerance under the conditions of this experiment, two multivariate ranking methods—Membership Function Value (MFV) and comprehensive PCA score (Fig. 4)—were employed. Both approaches showed a high degree of concordance and indicated a consistent tolerance hierarchy: X9 > C13 > S12 > S19 > C9 > JK. The MFV approach revealed clear genotypic variation, with D values ranging from 0.161 to 0.823. Within this dataset, X9 ranked highest (0.823), indicating relatively superior physiological and biochemical resilience under salinity stress, followed by C13 (0.675) and S12 (0.579). In contrast, JK (0.161) and C9 (0.276) recorded the lowest values, reflecting higher sensitivity under the tested conditions. The comprehensive PCA scores were strongly consistent with the MFV results, with X9 achieving the highest positive score (4.21) and JK the lowest negative score (-4.21), highlighting the contrasting responses among genotypes. Based on the integration of these results, the genotypes could be grouped into three categories under the present experimental conditions: tolerant (X9 and C13), moderately tolerant (S12 and S19), and sensitive (C9 and JK), corresponding to their relative physiological and biochemical performance. The strong agreement between these two independent methods suggests that the observed differences represent genuine biological variation in salinity tolerance within this study. However, further validation under different environmental conditions would be required to assess the broader applicability of this classification (Fig. 5).

#### Ultrastructure

**Fig. 6:**
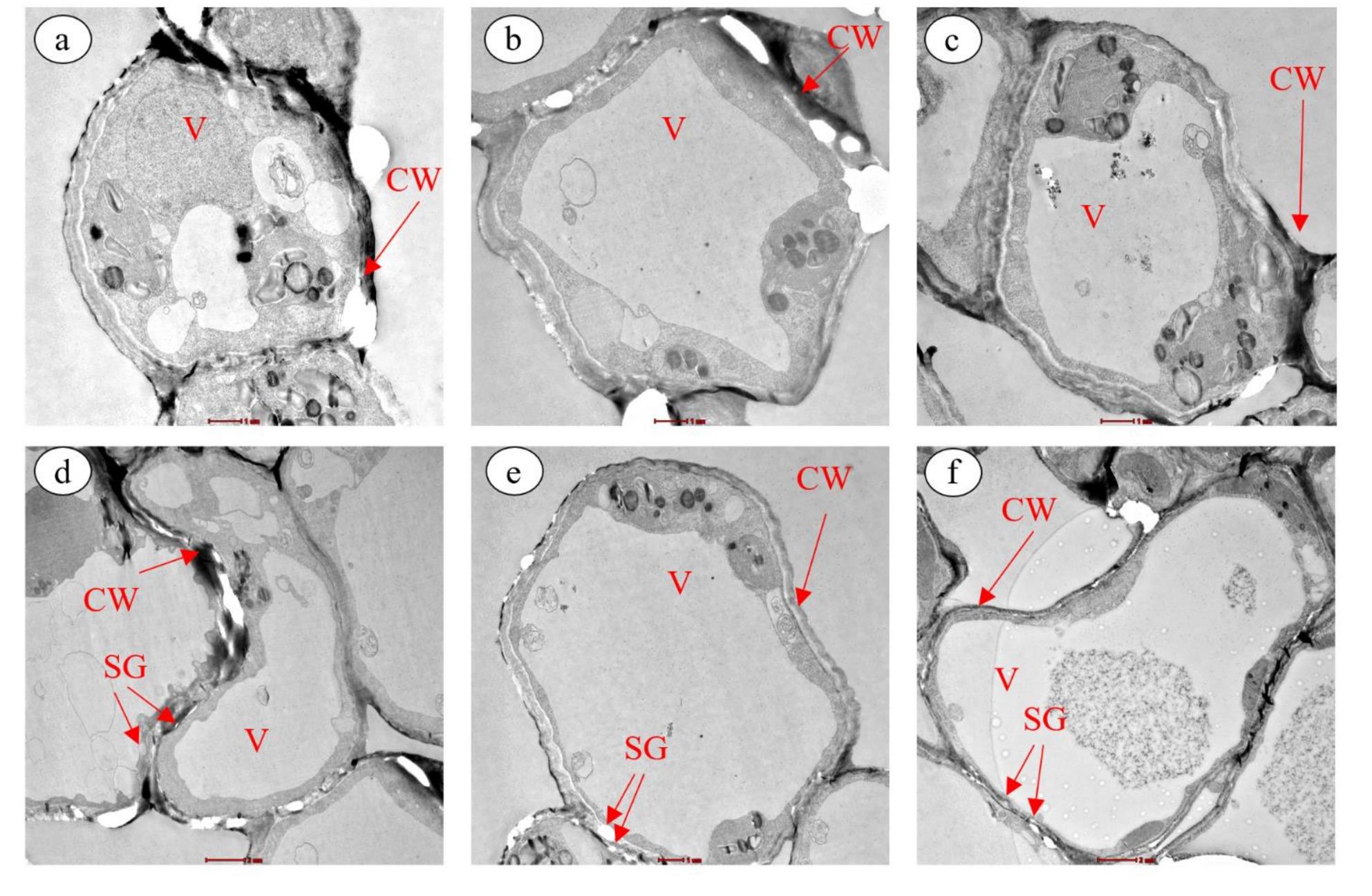
Transmission electron micrographs of mesophyll cell chloroplasts in citrus rootstocks SCSH 17 − 12 (S12), SCSH 9–19 (S19) and CRH 21 − 13 (C13) under control and salinity stress conditions. Scale bar = 2 μm. **a** SCSH 17 − 12, control; (**b**) SCSH 9–19, control; (**c**) CRH 21 − 13, control; (**d**) SCSH 17 − 12, salinity stress; (**e**) SCSH 9–19, salinity stress; (**f**) CRH 21 − 13, salinity stress

**Fig. 7:**
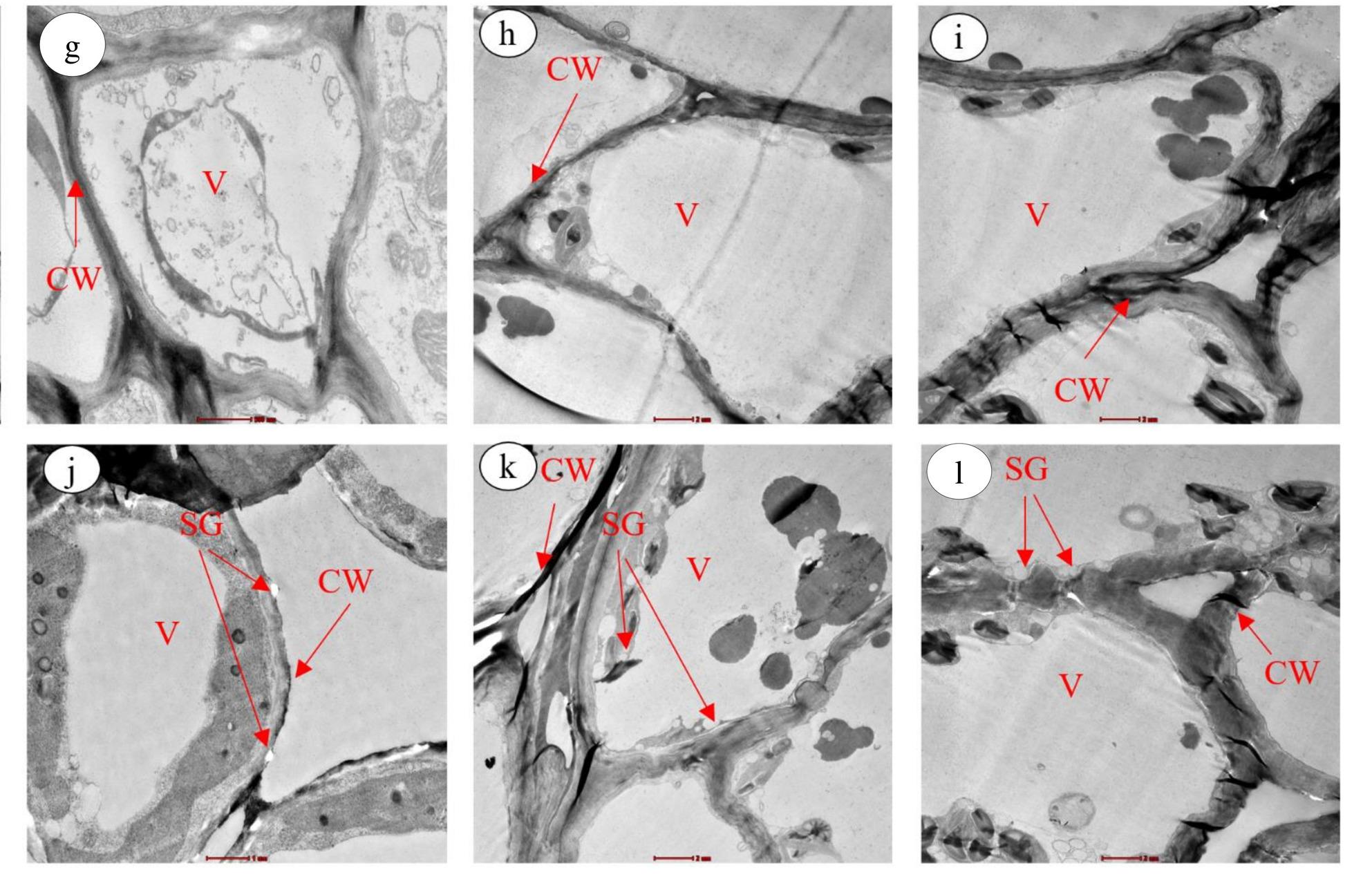
Transmission electron micrographs of mesophyll cell chloroplasts in citrus rootstocks X-639 (X9), CRH 21-9 (C9) and Jatti Khatti (JK) under control and salinity stress conditions. Scale bar = 2 µm. **g** X-639, control; (**h**) CRH 21-9, control; (**i**) Jatti Khatti, control; (**j**) X-639, salinity stress; (**k**) CRH 21-9, salinity stress; (**l**) Jatti Khatti, salinity stress

Salinity stress significantly altered chloroplast ultrastructure in citrus rootstocks, with the magnitude of change varying considerably among genotypes for chloroplast area, starch grain number and plastoglobuli number (Table 4) (Figs. 6 and 7).

#### Chloroplast area

**Fig. 8:**
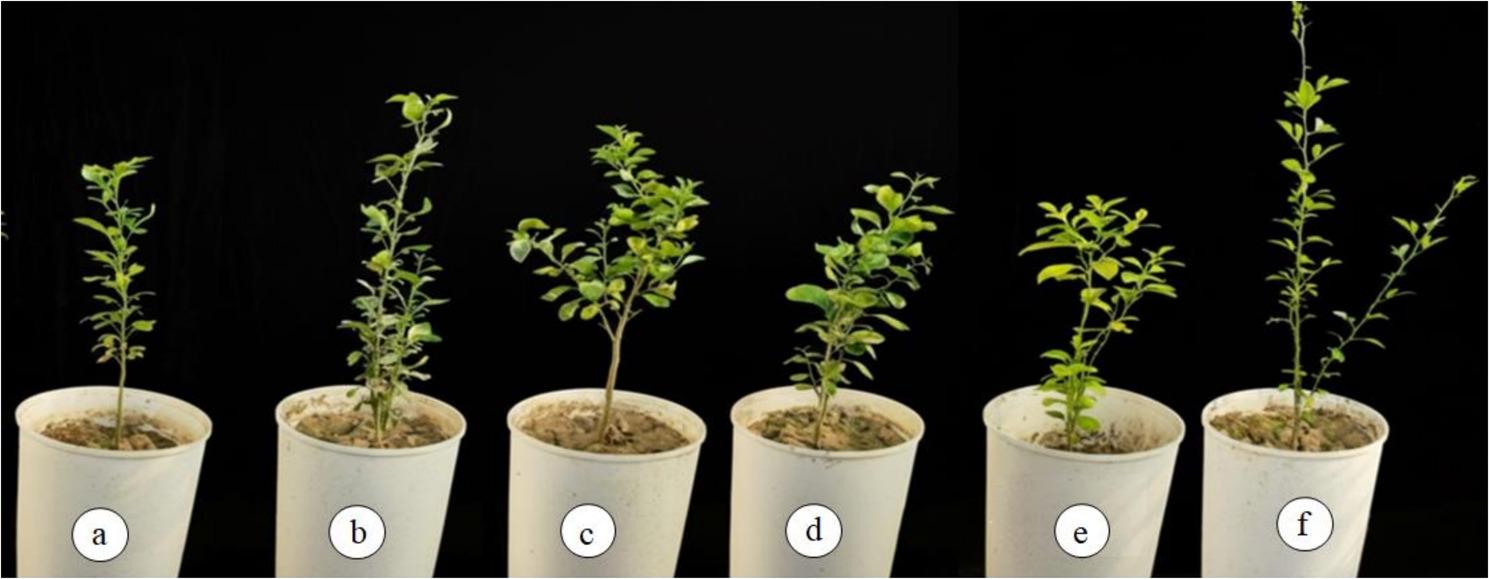
Phenotypic appearance of six citrus rootstocks under control conditions (0 mM NaCl). **a** CRH 21-9; (**b**) CRH 21-13; (**c**) SCSH 9-19; (**d**) SCSH 17-12; (**e**) Jatti Khatti; (**f**) X-639. All genotypes showed normal growth with healthy green leaves and no visual signs of stress, indicating that the variations observed under salinity stress (Fig. 8) are specifically due to differential salt tolerance responses among the genotypes

**Fig. 9:**
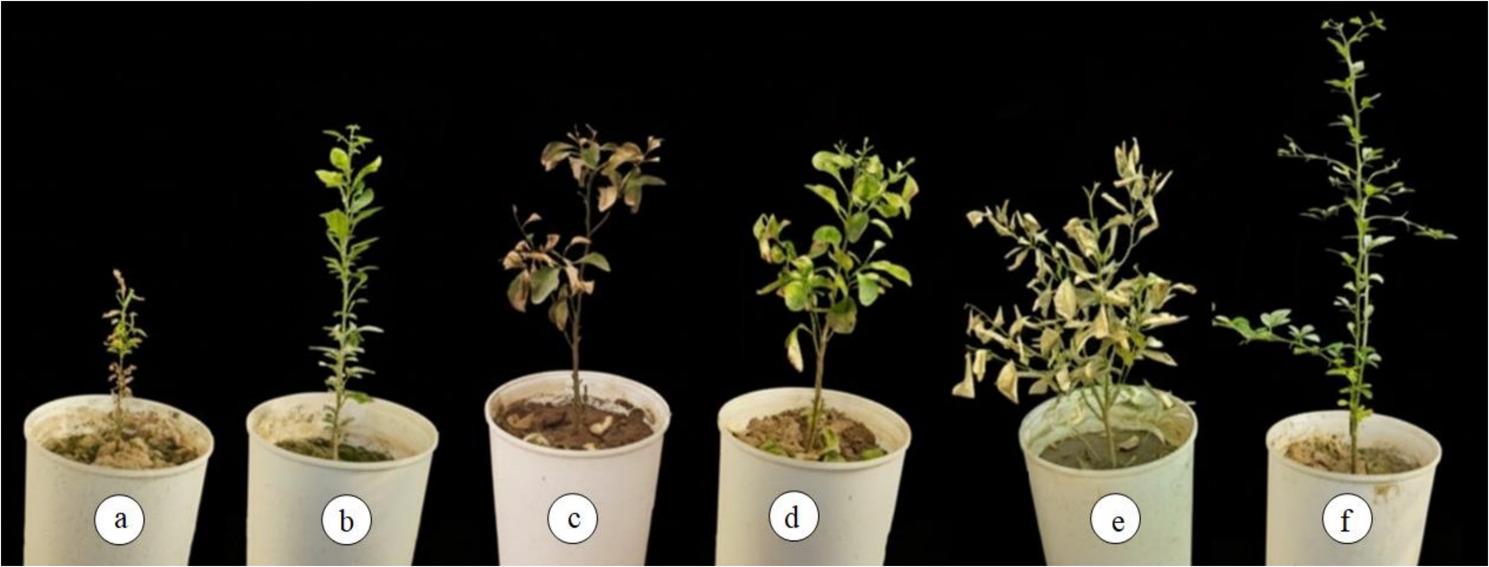
Photographs showing the effect of salinity stress (50 mM NaCl) on the growth and phenotypic appearance of six citrus rootstocks. **a** CRH 21-9; (**b**) CRH 21-13; (**c**) SCSH 9-19; (**d**) SCSH 17-12; (**e**) Jatti Khatti; (**f**) X-639. Tolerant genotypes (CRH 21-13, SCSH 17-12, X-639) maintained better growth with minimal leaf chlorosis and necrosis under salinity stress. In contrast, sensitive genotypes (CRH 21-9, SCSH 9-19, Jatti Khatti) exhibited severe growth reduction, pronounced leaf yellowing, wilting and tissue necrosis, reflecting their poor physiological and biochemical adaptation to salinity

Under control conditions, chloroplast area ranged from 8.3 ± 0.5 μm² in S19 to 8.7 ± 0.5 μm² in S12. Exposure to salinity led to a significant increase across all genotypes, with values rising to a range of 8.8 ± 0.5 to 11.2 ± 0.9 μm². JK exhibited the largest chloroplast area under salinity stress (11.2 ± 0.9 μm²), followed closely by C9 (10.8 ± 0.8 μm²) and S19 (10.1 ± 0.7 μm²). In contrast, C13 (8.8 ± 0.5 μm²) and X9 (8.9 ± 0.4 μm²) showed the smallest chloroplast areas under saline conditions, with values similar to S12 (9.3 ± 0.5 μm²). The percentage increase ranged from 4.7% in X9 to 33.3% in JK. While JK, C9 (28.6%) and S19 (21.7%) exhibited pronounced increases (> 20%), genotypes such as X9, C13 (4.8%) and S12 (6.9%) showed minimal changes (< 7%), indicating relative stability in chloroplast morphology (Figs. 8 and 9).

#### Starch grain number

Starch grain number per chloroplast ranged from 3.2 ± 0.3 in S12 and S19 to 3.4 ± 0.4 in JK, with all genotypes similar under control conditions. Salinity stress significantly reduced starch grain number across all genotypes, with values decreasing to a range of 0.8 ± 0.2 to 2.4 ± 0.3. X9 maintained the highest starch grain number under salinity stress (2.4 ± 0.3), followed closely by C13 (2.3 ± 0.3), with both similar to each other. Genotype S12 (1.9 ± 0.3) showed an intermediate reduction. The lowest starch grain numbers were observed in JK (0.8 ± 0.2) and C9 (0.9 ± 0.2), which were at par with each other, followed by S19 (1.2 ± 0.2). The percentage decrease ranged from 27.3% in X9 to 76.5% in JK. While X9, C13 (30.3%) and S12 (40.6%) showed moderate reductions (< 41%), genotypes such as JK, C9 (72.7%) and S19 (62.5%) exhibited pronounced decreases (> 60%), indicating greater sensitivity in terms of starch metabolism.

A significant G×S interaction (*P* ≤ 0.05) indicates variation in Chloroplast Area and Starch Grain Number responses among genotypes (Table 4).

## Discussion

### Photosynthetic pigments

#### Chlorophyll and photosynthetic machinery

Salt stress progressively depleted chlorophyll pigments across all the genotypes, though the magnitude of decline varied significantly among the studied rootstocks under salt stress [48]. Reductions in levels of Chl a suggest that the damage to PSII reaction centres is caused by ROS. These ROS likely target the D1 protein, causing disruption of the photosynthetic electron transport chain [49]. The tolerant genotypes X9 and S12 exhibited superior pigment retention, suggesting enhanced antioxidant protection and effective ion exclusion, which collectively preserve chloroplast integrity [22, 48].

Chl b and Tchl followed a similar pattern, although the level of reduction differed across genotypes. This pattern suggests that light-harvesting complexes are structurally sensitive to salinity, due to thylakoid membrane disorganization [32, 49–51].

Variations in pigment retention among genotypes reflect differences in their capacity for ion exclusion and antioxidant defense. The tolerant genotypes retained higher chlorophyll levels, accumulated less leaf Na⁺ and exhibited increased antioxidant enzyme activities. This confirms that chlorophyll preservation depends on multiple coordinated mechanisms, including limiting Na⁺ accumulation into chloroplasts, efficient scavenging of ROS and preserving the thylakoid membrane integrity. [51, 52]. The positive correlation between chlorophyll factions and both the K +/Na + ratio and SOD activity provides validation for this integrated interpretation.

### Leaf gas exchange parameters

#### A

Salinity stress significantly reduced *A* across all the studied genotypes. However, tolerant genotypes such as X9 and C13 maintained relatively stable photosynthetic rates. This stability highlights effective ion regulation and strong antioxidant defense mechanisms of the genotypes under salt stress conditions [32, 53]. In contrast, sensitive rootstocks (JK) exhibited severe inhibition of photosynthesis. This decline indicates ionic stress–mediated damage to PSII function and inhibition of carbon assimilation [54, 55]. This decline corresponds to the elevated leaf and root Na⁺ levels in sensitive genotypes, indicating that ionic toxicity disrupts the photosynthetic machinery through multiple pathways [53]. This interpretation is reinforced by the positive correlation of photosynthetic rate (A) with both the Na⁺ levels and SOD activity, revealing that retention of the photosynthetic process depends on the integrated regulation of ionic balance and antioxidant capacity [30, 51, 56].

#### gₛ

Under salinity stress, *gₛ* and *E* exhibited a similar response (Table 2). While tolerant genotypes X9 and S12 maintained relatively stable *gₛ* and *E* activity, indicating efficient root-level ion exclusion of toxic ions, significantly improved osmotic adjustment and preserved hydraulic function that sustain gas exchange [20]. In contrast, sensitive genotypes (JK, S19 and C9) exhibited marked reductions, reflecting stress-induced stomatal closure to limit transpiration and restrict the toxic ion influx [25, 57]. However, this adaptive response restricts CO₂ diffusion, thereby directly contributing to photosynthetic inhibition [58].

#### Ci

Patterns of *Ci* (Table 2) indicated distinct limitations on photosynthesis among the genotypes due to NaCl stress. Sensitive genotypes showed increased *Ci* despite reduced stomatal conductance, indicating the presence of non-stomatal limitations. This suggests that ionic toxicity impaired chloroplast function significantly, where available CO₂ was not efficiently fixed due to Na⁺-induced disruption of Rubisco activity and oxidative damage to enzymes involved in the Calvin cycle [25, 59]. Conversely, stable *Ci* in tolerant X9 and C13 reflect effective ion exclusion and antioxidant defense that protect photochemical and biochemical functions, indicating that stomatal limitation remained primary while photosynthetic biochemistry remained functional [55, 60–62]. The positive correlation between *Ci*, leaf Na⁺ and H₂O₂ supports this interpretation. It states that ionic and oxidative stress are the major drivers of the observed biochemical impairment.

#### E

Salinity stress resulted in substantial reductions in *E* across all genotypes, mainly due to stomatal closure for minimizing water loss. However, the magnitude of decline varied, reflecting differences in tolerance. The relatively smaller reduction in tolerent genotypes indicates its ability to maintain stomatal function and hydraulic balance under stress, thereby supporting continued gas exchange to maintain plant metabolic activity.

These gas exchange responses indicate that superior salinity stress tolerance of rootstocks such as X9 and C13 is associated with the maintenance of stomatal function and hydraulic conductance. However, this promotes continued CO₂ assimilation, nutrient transport and metabolic stability under salinity stress [51, 63, 64]. The ability to maintain transpiration while avoiding excessive ion influx is a critical strategy that balances water conservation and continued carbon accumulation.

### Osmolyte accumulation and non-enzymatic antioxidants

Analysis of oxidative stress markers revealed a marked distinction among genotypes under salt stress.

#### H₂O₂

The tolerant genotypes like X9 and C13 exhibited minor H₂O₂ increases, indicating effective ROS scavenging through enhanced antioxidant enzyme activity [65, 66]. However, sensitive genotypes including JK and S19 showed substantial H₂O₂ accumulation, reflecting an insufficient antioxidant capacity to mitigate harmful oxidative stress. This pattern demonstrated an inverse correlation with antioxidant enzyme activities, where tolerant genotypes maintained higher SOD, CAT and APOX activities under salinity [67, 68]. This indicates that H₂O₂ accumulation is result of insufficient scavenging rather than increased production. The strong negative correlations between H₂O₂ and both CAT and APOX activities further validate this conclusion [69, 70].

#### MDA

Elevated MDA levels in JK and C9 indicate loss of membrane integrity as a consequence of lipid peroxidation [28]. The data further suggest a harmful feedback loop, where ROS-induced membrane damage impairs Na⁺ exclusion, as evidenced by the positive correlation between MDA and leaf Na⁺ content. In contrast, the tolerant genotype X9 largely avoided such damage, maintaining lower MDA levels as result of higher CAT and SOD activities [66]. This indicates effective cellular membrane protection, which is widely recognized as a key feature of rootstock for enhanced salinity stress tolerance.

 [65–67].

#### TSS

Under salt stress, the tolerant genotype X9 exhibited a significant accumulation of TSS, indicating a superior capacity for osmotic adjustment. This physiological response likely stems from the enhanced activity of key biosynthetic enzymes, such as sucrose-phosphate synthase [71, 72]. By decreasing osmotic potential, elevated sugar levels help maintain cellular turgor and protect essential metabolic functions [73–76]. Consequently, the sustained synthesis of these osmolytes emerges as a critical trait, directly contributing to improved osmotic homeostasis and overall stress resilience in tolerant genotypes [77–79]. The alignment of these findings with recent studies across diverse species [20, 80] underscores the fundamental role of efficient carbohydrate mobilization in plant adaptation to saline environments.

#### LPC

Under salinity stress, LPC accumulation in leaves is largely regulated by increased activity of biosynthetic enzymes such as pyrroline-5-carboxylate synthetase (P5CS), along with modulation of catabolic enzymes like LPC dehydrogenase (ProDH) [81]. The elevated LPC levels observed in X9 and C13 (1.8–2.1-fold higher than control; Table 2) indicate a greater capacity for LPC biosynthesis. This likely contributes to stress tolerance by supporting osmotic adjustment, stabilizing proteins and reducing oxidative damage [26]. These findings are consistent with previous reports in citrus and other plant species [29, 74, 82].

### TPh

Total phenolic compounds, acting as non-enzymatic antioxidants, increased markedly in S12 and X9 (1.6-fold), whereas sensitive genotypes showed comparatively lower accumulation [32, 83–85]. Phenolics contribute to salinity tolerance through several mechanisms, including direct scavenging of reactive oxygen species via their aromatic structures, metal ion chelation, membrane stabilization and protection against UV-induced damage [48, 86]. The moderate negative correlation between phenolic content and H₂O₂ levels supports their role in mitigating oxidative stress. However, the pattern of phenolic accumulation did not fully correspond with tolerance ranking; for instance, S12 exhibited the highest increase despite only intermediate tolerance. This indicates that phenolics function as part of a broader set of partially overlapping antioxidant defenses rather than acting in isolation [48, 73, 76].

These findings demonstrate that salinity tolerance relies on the coordinated accumulation of multiple protective compounds rather than the maximal induction of any single osmolyte or antioxidant. Genotypes including X9 and C13, the more tolerant rootstocks were characterized by lower levels of oxidative damage markers including (H₂O₂ and MDA), higher accumulation of osmolytes (TSS and LPC), upregulated protein synthesis (TSP) and moderate phenolic induction [32, 48, 86]. This integrated response supports the effective osmotic adjustment, maintaining cell turgor and metabolic activity while providing a robust and complementary antioxidant defense to the salinity stress [73, 76]. In contrast, sensitive genotypes exhibit either weak induction or poor coordination of protective mechanisms, leading to the higher of oxidative damage and progressive cellular dysfunction under NaCl stress [32, 48, 86].These findings are supported by the work of Khalid [73] and Danaeifar [76].

### Antioxidant enzyme activities

#### Primary antioxidant enzymes

##### SOD

Genotypic variation in salinity tolerance is strongly linked to SOD induction [62], as this enzyme catalyzes the dismutation of superoxide radicals (O₂⁻) into H₂O₂ and O₂, representing the first, rate-limiting step in ROS detoxification. Salinity-tolerant genotypes, such as C13 and X9 (Table 3), displayed pronounced SOD upregulation, efficiently scavenging superoxide at production sites (chloroplasts, mitochondria, peroxisomes) [87]. This activity prevents Fe-S enzyme inactivation, limits oxidative cascades and, together with downstream peroxidases, mitigates H₂O₂ accumulation, maintaining cellular redox homeostasis.

##### APOX

Upregulated activity of ascorbate peroxidase (APOX) in the salt-tolerant genotypes X9 and C13 highlights a specialized enzymatic defense mechanism operating within the ascorbate–glutathione cycle [48, 88, 89]. This cycle serves as the primary pathway for hydrogen peroxide (H₂O₂) detoxification in both the chloroplasts and cytosol, playing a critical role in maintaining cellular redox homeostasis under salinity stress [90]. APOX catalyzes the reduction of H₂O₂ to water using ascorbate as a specific electron donor; the resulting monodehydroascorbate is then regenerated through the cycle. The pronounced induction of APOX activity limits the accumulation of H₂O₂, thereby reducing the potential for hydroxyl radical formation. This protective mechanism helps safeguard the photosynthetic apparatus and enhances overall oxidative stress resilience [91]. A positive correlation between APOX activity and net photosynthetic rate further supports this conclusion. However, susceptible genotypes exhibited only minimal increase in APOX activity, highlighting less effective ROS management under saline stress conditions [92]. These findings are consistent with earlier reports by Naghashi [74] and Mohammed [75], corroborating the importance of APOX in salinity tolerance.

##### CAT

Under NaCl-induced salinity stress, the susceptible genotypes exhibited upregulated CAT activity relative to controls (Table 2). Elevated CAT activity under saline conditions reflects distinct strategies for H₂O₂ detoxification [93], as CAT primarily targets H₂O₂ generated in peroxisomes during photorespiration and fatty acid oxidation [30]. The marked induction observed in these relatively sensitive genotypes likely represents a compensatory response to increased photorespiration resulting from salinity-induced stomatal limitations and insufficient APOX activity, promoting alternative pathways for H₂O₂ scavenging [76]. While, this strategy proved inadequate, as evidenced by constantly increased H₂O₂ and MDA levels in these genotypes. These findings suggest that CAT induction alone cannot compensate for limited H₂O₂ removal capacity in chloroplastic and cytosolic compartments [94]. A moderate negative correlation between CAT and APOX activities supports this interpretation. These observations are consistent with earlier findings reported by Shankar [79] and Mohammed [75].

#### Supporting enzymes

##### GR

GR activity, as presented in Table 3, increased markedly in tolerant genotypes such as C13 and X9 under salinity stress, indicating an enhanced capacity to maintain cellular redox homeostasis [48, 95]. By sustaining the GSH/GSSG ratio, GR supports the efficient functioning of the ascorbate–glutathione cycle and facilitates hydrogen peroxide detoxification [96, 97]. The positive correlation observed between GR and ascorbate peroxidase (APOX) activities further confirms their coordinated role in ROS scavenging.

##### POX

POX activity exhibited a contrasting trend. In the tolerant genotype X9, moderate POX activity alongside strong APOX and GR responses suggests a greater reliance on the ascorbate–glutathione cycle for precise redox regulation [98]. In contrast, higher POX activity in susceptible genotypes such as S19 and C9 appears to represent a compensatory antioxidative response under elevated oxidative stress [74, 88]. This mechanism contributes to H₂O₂ scavenging and cell wall stabilization; however, the weaker correlation between POX activity and overall tolerance compared to APOX or SOD indicates that POX plays a secondary, context-dependent role in salinity tolerance [99].

The analysis of antioxidant enzyme profiles indicates that salinity tolerance is governed by the coordinated activation of complementary pathways [74, 75]. Tolerant genotypes such as X9 and C13 exemplify this balanced network through a multifactorial enzymatic strategy. This integrated coordination ensures efficient conversion and detoxification of reactive oxygen species while preserving the stress signaling functions necessary for acclimation.

### Leaf mineral nutrient homeostasis

#### Potassium and calcium

Our results highlight a stark contrast among rootstocks in their capacity to preserve leaf K⁺ and Ca²⁺ balance during salt exposure, a divergence that mirrors the decline we tracked in the gas exchange data. Sensitive lines, particularly S19 and JK, suffered a pronounced depletion of both cations that appears to stem from more than passive osmotic adjustment [86]. While the depolarization of root membranes and antagonistic competition with Na⁺ at transport sites make some loss of K⁺ predictable, the severity observed in S19 points to an underlying inefficiency in root retention mechanisms [100, 101]. The strong correlation we found between leaf K⁺ status and both stomatal conductance and net photosynthesis provides a clear functional bridge connecting this ionic leakage to the impairment of carbon assimilation [102, 103].

A parallel reduction in leaf Ca²⁺ carries implications that extend well beyond cell wall integrity and into the realm of cellular signaling. A diminished pool of available Ca²⁺ in the mesophyll likely attenuates the activation of the Salt Overly Sensitive (SOS) cascade, a pathway essential for mounting an effective defense. Without sufficient cytosolic Ca²⁺ to trigger the SOS3–SOS2 kinase complex, the phosphorylation and activation of the plasma membrane SOS1 Na⁺/H⁺ antiporter are presumably compromised, weakening the plant’s frontline defense against Na⁺ influx [104]. This may establish a detrimental feedback loop wherein the initial salt insult degrades Ca²⁺ signaling, thereby crippling the plant’s subsequent ability to exclude Na⁺. The tolerant genotype X9 distinguished itself by maintaining leaf K⁺ and Ca²⁺ at metabolically viable concentrations, a trait reflected in its remarkably high K⁺/Na⁺ ratio and suggestive of a root system with either superior transporter selectivity or robust membrane potential regulation under salinity stress [103]. We also noted a positive relationship between Ca²⁺ levels and superoxide dismutase (SOD) activity, hinting at a contributory role for this cation in priming the antioxidant machinery [20, 83–85, 105].

#### Magnesium dynamics

Leaf Mg²⁺ exhibited a similar trajectory of loss in the sensitive germplasm, a trend that carries immediate consequences for photosynthetic machinery. The depletion stems partly from ionic displacement at root exchange interfaces, yet the downstream ramifications in the leaf are profound given Mg²⁺’s central position in the chlorophyll porphyrin ring and its necessity for RuBisCO activation and ATP metabolism [67, 106]. Our correlation analysis confirmed this functional dependency, showing a tight link between Mg²⁺ status and sustained chlorophyll concentration. By contrast, X9 and to a lesser degree C13 demonstrated a capacity to buffer their leaf Mg²⁺ reserves against this decline. This protective effect likely arises from a combination of persistent root uptake activity and diminished apoplastic bypass flow in the root cortex, a feature that would limit the competition and displacement of Mg²⁺ from binding niches by Na⁺ ions moving within the transpiration stream [107].

#### Root ion dynamics as the mechanistic basis for shoot homeostasis

While leaf profiles reveal the shoot’s physiological experience, our root tissue analysis offers a mechanistic explanation for the observed contrasts in foliar nutrition. The additional measurements of root K⁺, Ca²⁺, and Mg²⁺ proved particularly instructive. The restrained shoot Na⁺ concentrations we documented in X9 were not achieved through wholesale root exclusion; instead, X9 roots actually harboured higher Na⁺ loads than the sensitive checks. This pattern indicates a proficient strategy of vacuolar containment within the root cortex, likely mediated by mechanisms such as tonoplast-bound NHX antiporters, effectively immobilizing the toxic ion and curtailing its entry into the xylem conduit [103]. By compartmentalizing Na⁺ stress within the root system, the plant shields its photosynthetic apparatus at a relatively modest energetic cost.

The root nutrient data further elucidate the origin of foliar depletion in S19 and JK. It was not merely a passive effect of growth dilution; these genotypes simultaneously exhibited a significant drop in their root K⁺ and Ca²⁺ pools. This finding suggests that the disruption of ionic equilibrium initiates at the root-soil boundary. It suggests a root plasma membrane that is uniquely susceptible to Na⁺, which triggered K⁺ efflux via channels such as GORK and vulnerable to the disruption of high-affinity transport systems responsible for K⁺ and divalent cation acquisition [102, 104–106, 108]. In stark contrast, X9 roots sustained markedly elevated levels of K⁺, Ca²⁺, and Mg²⁺ under identical external conditions. The capacity to uphold nutrient capture within a Na⁺-laden medium is a defining feature of tolerance, implying superior membrane integrity and likely enhanced development of apoplastic impediments like suberin lamellae or Casparian bands to thwart unregulated Na⁺ intrusion [20].

#### Integration and synthesis of mineral nutrient responses

Collectively, these mineral profiles indicate that salinity resilience in citrus rootstocks hinges on an integrated strategy rather than a singular genetic trait. The robust performance of X9 appears rooted in a coordinated policy of root-level Na⁺ sequestration to safeguard the shoot, coupled with the metabolic vigour needed to keep high-affinity K⁺ and Ca²⁺ transport operational despite the ionic challenge [51, 56]. This equilibrium sustains the internal milieu required for enzymatic activity and membrane stability within photosynthetic tissues. Conversely, sensitive genotypes appear ensnared in a systemic collapse wherein root ionic chaos starves the foliage of essential nutrients, leading to diminished photosynthetic output. This decline likely curtails the energy supply necessary to fuel active ion exclusion at the root, thereby perpetuating a vicious cycle of energy deficit and ion leakage that defines the stress response in S19 and JK.

In summary, the defining hallmarks of a robust rootstock in this study are a low foliar accumulation of Na⁺ and Cl⁻, a favorable K⁺/Na⁺ ratio, and the retention of leaf Ca²⁺ and Mg²⁺ at or above 80% of unstressed control levels [109–111]. X9 consistently satisfied these benchmarks, underscoring its promise both as a commercial rootstock and as a genetic donor. C13 occupies a middle ground with moderate regulatory efficiency, whereas the unfavorable ion profiles of JK and S19 limit their suitability for cultivation in salt-affected environments [112, 113].

#### Tolerance ranking

The tolerance ranking generated in this study indicates that X9 and C13 are among the most salt-tolerant citrus rootstocks currently available. When compared with commonly used rootstocks such as Troyer citrange and Cleopatra mandarin, X9 shows a clear physiological advantage. For instance, Na⁺ accumulation in X9 remains substantially lower, while the decline in photosynthetic activity is less severe under comparable stress conditions [114, 115]. Hybrids such as S12 and C13 exhibit responses closer to Cleopatra mandarin, suggesting moderate tolerance. In contrast, S19 and C9 behave more like Troyer citrange, showing higher sensitivity to salinity stress. This gradient in response highlights the genetic variability within the evaluated material. Overall, X9 and C13 emerge as promising candidates for saline environments, offering a practical improvement over currently used commercial rootstocks [116]. The enhanced salinity tolerance observed in X9 appears to arise from a tightly coordinated regulatory system that integrates ion exclusion, antioxidant defense and osmotic adjustment [117, 118]. At the physiological level, this genotype maintains low Na⁺ and Cl⁻ accumulation along with a high K⁺/Na⁺ ratio, suggesting more efficient activity of key ion transporters involved in ion homeostasis [119]. As a result, ionic toxicity is minimized and the plant avoids excessive activation of stress signalling pathways that would otherwise compromise growth.

Genotype X9 showed strong induction of antioxidant enzymes such as SOD, APOX and GR, indicating an efficient ROS detoxification system under salinity stress [120, 121]. This response likely protects cellular membranes and metabolic processes from oxidative damage. In addition, higher accumulation of osmolytes such as LPC and TSS contributes to osmotic adjustment, helping maintain cellular turgor and metabolic stability under reduced water potential [122]. Together, these responses reflect a coordinated defense strategy involving effective ion regulation, controlled oxidative stress and maintenance of cellular homeostasis. Similar integrated mechanisms have been reported in model systems such as Arabidopsis and tomato, where WRKY transcription factors and the SOS signalling pathway (SOS1, SOS2, SOS3) regulate ion homeostasis [123].

In contrast, salt-sensitive genotypes such as S19 and JK exhibited excessive Na⁺ and Cl⁻ accumulation, indicating weaker ion regulation at the root level. This may be associated with reduced efficiency of key transporters involved in ion exclusion and compartmentalization, including SOS1, HKT1 and NHX1 [124–126]. The resulting ionic imbalance, coupled with limited antioxidant induction, likely leads to increased oxidative damage and impaired photosynthesis [127]. However, these interpretations are based on indirect evidence and require validation through targeted molecular approaches such as qRT-PCR and protein expression analyses.

#### Microscopy

Ultrastructural analysis revealed clear genotype-dependent alterations in chloroplast morphology under salinity stress, providing cellular-level evidence that supports the observed physiological and biochemical responses. Changes in chloroplast area and starch grain number served as reliable indicators of chloroplast integrity and closely corresponded with the tolerance ranking [128, 129]. Sensitive genotypes (JK, C9 and S19) showed pronounced chloroplast swelling, likely due to Na⁺ and Cl⁻ accumulation, reflecting osmotic imbalance and disruption of organelle homeostasis [47, 128]. In contrast, tolerant genotypes (X9, C13 and S12) maintained near-normal chloroplast structure, suggesting effective ion regulation, osmotic adjustment and protection of thylakoid organization. This structural stability was associated with better photosynthetic performance, whereas swollen chloroplasts in sensitive genotypes were linked to impaired electron transport and reduced carbon assimilation [128, 129]. Starch grain depletion further indicated metabolic disruption under salinity stress, particularly in sensitive genotypes, where reduced photosynthesis and increased energy demand led to depletion of carbon reserves [130]. Strong positive relationships among starch content, photosynthetic rate and soluble sugars suggest coordinated carbon metabolism in tolerant genotypes [129]. Together, chloroplast swelling and starch depletion represent a linked damage response driven by ionic and oxidative stress [128]. In contrast, tolerant genotypes, especially X9, mitigated these effects through integrated mechanisms involving ion homeostasis, enhanced antioxidant capacity and sustained photosynthetic activity, thereby preserving chloroplast structure and metabolic function under saline conditions [129, 130].

### Multivariate insights into salinity tolerance mechanisms

#### PCA biplot

Multivariate analysis provided a more integrated view of salinity responses by revealing the interaction of physiological and biochemical traits. The PCA biplot highlighted a clear trade-off along the primary axis (PC1) between photosynthetic parameters and toxic ion accumulation, confirming that Na⁺ and Cl⁻ buildup is a central constraint on carbon assimilation in these genotypes [29, 131]. The second component (PC2) captured variation in biochemical defense capacity that was independent of the primary physiological decline. This axis was strongly associated with APOX, TPh and SOD [66, 72, 132]. The distinct upward positioning of X9 and C13 along PC2 suggests a more effective activation of ROS scavenging mechanisms. However, the limited separation of S19 and JK along this axis indicates that their sensitivity was due to poor ion exclusion and a comparatively weak antioxidant response.

#### Hierarchical clustering

Hierarchical clustering reinforced the grouping of tolerant genotypes with traits related to photosynthetic maintenance and osmolyte accumulation (TSS, TSP), while sensitive genotypes clustered with oxidative stress markers such as MDA and H₂O₂, along with elevated CAT activity. The co-occurrence of high CAT and H₂O₂ in these genotypes suggests a compensatory but insufficient detoxification response, where ROS production exceeds scavenging capacity, leading to membrane damage [66, 72, 132].

#### Correlation analysis

Correlation analysis further reinforced these interactions. A strong positive association between Cl⁻ and MDA (*r* > 0.9; Fig. 2) indicates that chloride toxicity is a major driver of membrane lipid peroxidation in these genotypes [29, 133, 134]. Additionally, the inverse relationship between CAT activity and net photosynthesis suggests a metabolic trade-off, where increased investment in ROS detoxification constrains carbon assimilation. In contrast, X9 maintained high photosynthetic performance alongside active antioxidant metabolism, indicating a more efficient coordination between defense and growth processes [111, 135].

While the physiological and biochemical data presented here provide strong evidence for differential salt tolerance mechanisms, molecular confirmation such as gene expression analysis of ion transporters (SOS1, HKT1, NHX1, HAK/KUP), antioxidant enzymes (SOD, APOX, CAT) and signaling components (SOS2, SOS3, WRKY), was beyond the scope of this study. The mechanistic interpretations offered should therefore be considered testable hypotheses requiring molecular validation in future investigations.

## Conclusions

This study demonstrates that salinity tolerance in citrus rootstocks is the result of the coordinated interaction of multiple complex mechanisms. Multivariate analysis under the present experimental conditions indicated a consistent tolerance ranking: X9 > C13 > S12 > S19 > C9 > JK. The relatively superior performance of X9 was associated with four key attributes: effective ion exclusion, strong antioxidant defense, enhanced osmotic adjustment, and preservation of chloroplast ultrastructure. Transmission electron microscopy further confirmed that chloroplast damage closely corresponded with the degree of physiological impairment, as observed in the genotypes under control and salinity conditions. These results provide practical criteria for selecting citrus rootstocks under saline conditions, including low shoot Na⁺, a high K⁺/Na⁺ ratio, strong antioxidant enzyme activity (particularly SOD and APOX) and maintenance of chlorophyll content. Among the evaluated genotypes X9 followed by C13, emerge as the most promising salt-tolerant rootstock for cultivation in salt-affected environments. Although this study offers a comprehensive physiological and biochemical characterization, it was conducted under controlled greenhouse conditions at the seedling stage. Future studies are needed to further validate these findings under field conditions, investigate rootstock–scion interactions and employ transcriptomic approaches to identify the genetic basis of the observed tolerance mechanisms.

## Data Availability

The data that support the findings of this study are available from the corresponding author upon reasonable request.
